# Supplementary material for: Origin and dispersal history of Hepatitis B virus in Eastern Eurasia
Source: Nat Commun. 2024 Apr 5;15:2951. doi: 10.1038/s41467-024-47358-6 (PMC10997587; doi:10.1038/s41467-024-47358-6)
Supplement: Supplementary file 1 — Supplementary Information [file 41467_2024_47358_MOESM1_ESM.pdf]

Supplementary Information

Origin and dispersal history of Hepatitis B virus in Eastern Eurasia

Bing Sun[1]†, Aida Andrades Valtueña[2]†, Arthur Kocher[2,3], Shizhu Gao[4], Chunxiang Li[1], Shuang Fu[1], Fan Zhang[1], Pengcheng Ma[1], Xuan Yang[1], Yulan Qiu[1], Quanchao Zhang[5], Jian Ma[6], Shan Chen[7], Xiaoming Xiao[7], Sodnomjamts Damchaabadgar[8], Fajun Li[9], Alexey Kovalev[10], Chunbai Hu[11], Xianglong Chen[12], Lixin Wang[13], Wenying Li[14], Yawei Zhou[15], Hong Zhu[13], Johannes Krause[2*], Alexander Herbig[2*], Yinqiu Cui[1*]

* Correspondence to: Yinqiu Cui (cuiyq@jlu.edu.cn), Johannes Krause (krause@eva.mpg.de) and Alexander Herbig (alexander_herbig@eva.mpg.de)

This file includes:

Section 1: Genome list for mapping
Section 2: Archaeological background
Section 3: Figs. S1 to S10
Section 4: Tables. S1 to S3

Section 1: Genome list for mapping

Reference list of mapping for each lineage

Lineage	Reference (Unique record identifiers for NCBI)
A	FJ692557
B	D23679.1
C	GQ377617.1
D	FJ692507.2
WENBA	AF222323.1

Genome list of genotypes for comparative mapping

Genotype	Reference (Unique record identifiers for NCBI)
A	FJ692557
A	X02763
B	GU815637
C	GQ377617
D	KC875277
D	NC_003977
E	GQ161817
F	HM585194
G	AB056513
H	FJ356715
I	AB562463
J	AB486012
Gibbon	FM209516.1
Gorilla	AJ131567.1
OG	AF193863.1
Chimp	AF222323.1

We select these genomes as our reference sequences based on a study that provides a comprehensive set of HBV reference sequences at both the genotype and subgenotype levels[1]. One reason we decided not to use ancient sequences as reference is that the modern genome we selected are complete, whereas the ancient sequences available often lack comprehensive coverage.

Section 2: Archaeological background

Tsagaan Del Historical period burials in Dornogovi Province, Mongolia (MY12, MY17, MY19)

This site was excavated in Tsagaan Del mountain, Dornogovi Province, Mongolia, including 24 burials. Based on the burial structure and funerary objects these burials were attributed to the 13th-14th century, the late Mongol Empire to the Yuan dynasty. In this study, a total of 24 ancient individuals from the site were screened.

◆ **MY12: 715 ± 20 cal. a B.P.**

XiHe Early Iron Age burials in Hebei Province, China (XHM12, XHM16, XHM18, XHM23, XHM31)

XiHe cemetery is located in Hebei province, China. Based on funerary objects and archaeological studies these burials were attributed to Spring and Autumn Warring States Period (770 BC – 221 BC). In this study, a total of 41 ancient individuals from the site were screened.

◆ **XHM18: 2440 ± 25 cal. a B.P.**

NanYang Early Iron Age burials in Hebei Province, China (NYM8)

NanYang cemetery is located in Heibei province, China, nearby XiHe cemetery. Based on funerary objects and archaeological studies these burials were attributed to Spring and Autumn Warring States Period (770 BC – 221 BC). In this study, one ancient individual from the site were screened.

Bayanbulag (Baian Bulag) Early Iron Age burials in Umnugovi Province, Mongolia (AT7, AT19, AT24,)[2]

Bayanbulag (Baian Bulag) is located in Umnugovi (South Gobi) Province, Mongolia. In Mongolian, the name "Baian Bulag" means "rich spring". It may be reflected here is a suitable place for agriculture. It was first discovered in 1957 by Kh. Perlee. The archaeological research of the Baian Bulag began in 1957 when Kh. Perlee undertook an archaeological survey of the territory of Nomgon sum [3]. After that, it has been excavated three times in 1976, 1990, and 2009.

20 skeletons and 33 body fragments were found in an irregular round pit of approximately 7 meters in diameter and 1.3 meters deep at this site. According to the features of their relatively complete skeletons, 17 of these individuals can be determined as male adults aged between 20-25 years old (DOI: 10.1111/arcm.12556). The spine of the individual AT19 had been broken in the lumbar portion; judging from the signs of cleaving at the vertebrae and the lack of the lower portion of the body, the individual had been cut in half.[3] Two men, AT5 and AT7, lay kneeling with their feet under the pelvis. It may indicate that they were on their knees when they had been killed, which has been interpreted as execution of people that had been captives[3].

There are fragments of numerous Chinese-style vessels have been found here, such as "pen"-basins, "fu"-cauldrons, "guan"-jars, "zeng"-steamers, "weng"-pots, and, as was mentioned before, one leg of a huge tripod "ding"-cauldron[3]. They also found lots of weapons, such as iron knife fragments, pieces of iron "fu"-cauldrons, iron "Ji"-halberd, iron armor plates, and Bronze arrowheads.[3]

In this study, a total of 23 ancient individuals from the site were screened. All individuals from this site were buried during the same period. Consequently, the dating information for individuals AT6 and AT8 from the site can serve as a reference for the age information of HBV-carrying individuals. The radiocarbon dating results for these two individuals are as follows:

- **AT6: 2200 ± 30 cal. a B.P.**[2]
- **AT8: 2160 ± 30 cal. a B.P.**[2]

TaoJiaZhai Early Iron Age burials in Qinghai province, China (TJZM25_2)

TaoJiaZhai tomb is located in Qinghai province, China. It was first excavated by the Qinghai Provincial Institute of Archaeology and Xining Municipal Institute of Cultural Relics Management between the 1980s and 1990s. This first excavation consisted in a small-scale excavation, which was followed by a large-scale excavation led by the Qinghai Provincial Institute of Archaeology between 2002 and 2006, yielding 89 burials. Based on archaeological studies these burials were attributed to Han and Jin dynasties (1700-1900 years ago)[4]. One individual tested positive for HBV and was AMS dated. In this study, a total of 22 ancient individuals from the site were screened.

- **TJZM25_2: 1765 ± 30 cal. a B.P.**

QuanErGou Late bronze age burials in Xinjiang, China (XBQM20, XBQM46, XBQM47, XBQM86, XBQM125)

QuanErGou cemetery is located in the Eastern Tianshan Mountains, Uygur Autonomous Region of Xinjiang, China, which is a public cemetery of the ancient nomadic population. Excavation work was undertaken by the Xinjiang Institute of Archaeology. Archaeological studies suggest that QuanErGou cemetery is from the Bronze Age period. As part of the Silk Road and located at the geographic confluence of Eastern and Western cultures, Xinjiang served as a major crossroads. Five individuals tested positive for HBV and two were AMS dated. In this study, a total of 44 ancient individuals from the site were screened.

- **XBQM46: 2885 ± 25 cal. a B.P.**
- **XBQM125: 2895 ± 25 cal. a B.P.**

Derestuj Early Iron Age burials in Irkutsk Oblast, Russia (XN12)

Derestuj burial was discovered by J. Talko-Gryntsevich in the Trans-Baikal area near the Russian-Mongolian border during 1896-1902. Archaeologists confirmed that the site belonged to Hsiung-nu and this site has yielded very important evidence for Hsiungnu's social history. A large number of artifacts were found in the graves, the most common being belts and other details of clothing, (principal plates made in the Ordos style), ceramics, glass stone beads, etc. The belts consisted of a central part (normally two bronze plates) and other details like small bronze and stone plates, bronze, iron, and stone rings, fasteners, etc. Scenes in the Ordos style are represented on the bronze plates - for example fighting horses, a beast of prey grasping a herbivorous animal, and fighting dragons. One individual tested positive for HBV and was AMS dated. In this study, a total of 13 ancient individuals from the site were screened.

- **XN12: 2060 ± 20 cal. a B.P.**

XingFuLinDai Historical period burials in Shanxi Province, China (XFLDM173)

XingFuLinDai cemetery is located in Xian, Shanxi Province, China, and has been excavated by the Xian Institute of Cultural Relics Protection and Archaeology in 2017. The excavation finally yielded 2061 skeletal remains. Based on archaeological studies these burials were attributed to the middle of the Tang dynasty (800 years ago). In this study, a total of 9 ancient individuals from the site were screened.

LongTouShan Late bronze age burials in Inner Mongolia, China (91KLH18)

LongTouShan burials are located in Chifeng, West Liao (WLR) River region, northeast China This site has been first discovered in 2008 and excavated three times in 1987, 1988, and 1989 by the institute of Cultural Relics of Inner Mongolia. This site was attributed to nomadic pastoralism in the Upper Xiajiadian culture (1000–600 BCE) according to the archaeological background. The individual (91KLH18) infected with HBV has been AMS dated before[5] In this study, a total of 19 ancient individuals from the site were screened.

- **91KLH18: 2717±30 cal. a B.P.** [5]

FuLuTa Early Iron Age burials in, China (FLTM18, FLTM48, FLTM97, FLTM101)

FuLuTa cemetery was first founded in Inner Mongolia, China in the 1980s. Then the Institute of Cultural Relics and Archaeology of Inner Mongolia Autonomous Region excavated this cemetery in 2017. A total of 32 rectangular shaft-pit tombs were found. More than 200 artifacts, including ceramics, bronzes, iron, jade, stoneware, bone tools, and glasses, were discovered. Based on the cultural characteristics of these remains, this cemetery was assumed to date from the Late Warring States to the Early Western Han periods, and belongs to the Qin culture.[6] We found genomic evidence of HBV in two individuals from this cemetery. Both of them have been AMS dated in this study. In this study, a total of 56 ancient individuals from the site were screened.

- **FLTM48: 2130 ± 25 cal. a B.P.**
- **FLTM101: 2130 ± 20 cal. a B.P.**

JiangJiaLiang Late Neolithic burials in Henan Province, China (98JJLM9, 95JJLM34, 95JJLM51)

JiangJiaLiang Late cemetery is located in Yuanyang, Henan Province, China, and has been excavated by the Henan Provincial Institute of Cultural Relics twice in 1995 and 1998. This site is in the period of transition from Yangshao culture to Longshan culture. A total of 78 burials were found here and artifacts including pottery, millstones, stone axes, and flake tools were excavated from these burials[7]. Three individuals tested positive for HBV and have been AMS dated in this study. In this study, a total of 44 ancient individuals from the site were screened.

- **98JJLM9: 4065 ± 25 cal. a B.P.**
- **95JJLM34: 4130 ± 25 cal. a B.P.**
- **95JJLM51: 4130 ± 25 cal. a B.P.**

QiLangShan Historical period burials in Inner Mongolia, China (ZQM16)

QiLangShan burials were located in Inner Mongolia, China, and have been excavated by the Inner Mongolia Institute of Cultural Relics and Archaeology, Ulanqab Museum between 1995 and 1996. A total of 20 burials were been found, showing a close relation between Xianbei in cultural characteristics. One individual tested positive for HBV and was AMS dated in this study. In this study, a total of 6 ancient individuals from the site were screened.

- **ZQM16: 1580 ± 25 cal. a B.P.**

Northern Early Middle Bronze age burials in Xinjiang, China (11KBM13)

Northern Cemetery (Beifang cemetery) is located in Taklamakan, Xinjiang, China has been

discovered by an Uyghur peasant wandering in the desert in 2008. The excavation works were carried out by the Xinjiang Uyghur Autonomous Region (XUAR) Institute of Archaeology. This cemetery is in the southwest of Xiaohe cemetery, 600 km far from Xiaohe cemetery, and share many cultural affinities to the Xiaohe culture complex[8]. Five samples were AMS dated between 1800 BC and 1500 BC in the study [8]. Here, we found one individual was infected with HBV and was AMS dated in this study. In this study, a total of 11 ancient individuals from the site were screened.

- **11KBM13: 3405 ± 30 cal. a B.P.**

Honghe Historical period burials in Heilongjiang, China (HHM29)

The Honghe site is located in Qiqihar city, Heilongjiang, China. In 2004 and 2009, the cultural heritage department of Qiqihar conducted basic clean-ups of two exposed tombs at the Honghe site. Between June and November of 2013, the Heilongjiang Provincial Institute of Cultural Relics and Archaeology carried out the first excavation of the Honghe site. The site encompasses relics from three different periods: the Ming and Qing dynasties, the Bronze Age, and the Neolithic period. In this study, a total of 44 ancient individuals from the site were screened.

Hengshui Early Iron Age burials in Shanxi, China (JHM2098)

The Hengshui site, located in Yuncheng City, Shanxi Province, is a burial site of the Peng State, a feudal state during the middle period of the Western Zhou Dynasty. From December 2004 to December 2007, the Shanxi Provincial Institute of Archaeology conducted extensive excavations. A total of 1,326 tombs were excavated, of which 1,299 span from the later part of the early Western Zhou to the end of the Western Zhou period. In this study, a total of 56 ancient individuals from the site were screened.

Niuheliang Late Neolithic burials in Liaoning, China (96NVZIM6)

The Niuheliang site is a significant group of sites from the late Hongshan culture of the Neolithic period in northern China, located in Jianping County, Chaoyang City, Liaoning Province. The site dates back to approximately 5,500 to 5,000 years ago. It was discovered during a cultural relics survey in 1981. Excavations began in 1983 under the leadership of the Liaoning Provincial Institute of Cultural Relics and Archaeology. The Niuheliang site complex includes: the Goddess Temple, altars, stone cairns, and pyramids. In this study, a total of 21 ancient individuals from the site were screened.

TianTaiJie Historical period burials in Shanxi, China (SBSM101)

The TianTaiJie Cemetery is located in Datong City, Shanxi Province, and was the site of Pingcheng, the capital during the middle period of the Northern Wei Dynasty. Pingcheng was the political, economic, and cultural center of the Northern Wei. The Northern Wei was a regime established by the Xianbei people. The Datong Archaeological Research Institute conducted the initial excavation of the site in 2015, unearthing skeletal remains from 116 tombs dating back to the Northern Wei period. In this study, a total of 50 ancient individuals from the site were screened.

Shirenzigou Early Iron Age burials in Xinjiang, China

The shirenzigou site is located in the Hami region of the Xinjiang Uyghur Autonomous Region. It preserves cultural remains spanning from the Bronze Age to the Sui-Tang period. In 2006, the

Faculty of Cultural Heritage of Northwest University collaborated with the Xinjiang Archaeological Research Institute to conduct archaeological excavations here. Among the site, five samples have radiocarbon dating data, all of which belong to the Iron Age. The results are as follows. In this study, a total of 16 ancient individuals from the site were screened.

- **SRZGM4: 2290 ± 90 cal. a B.P.**
- **SRZGM8R1: 2265 ± 65 cal. a B.P.**
- **SRZGM820: 2252 ± 22 cal. a B.P.**
- **SRZGM010: 2380 ± 30 cal. a B.P.**
- **SRZGF004: 2280 ± 90 cal. a B.P.**

Houtao Neolithic burials in Jilin, China

The Houtao site is located in Da'an City, Baicheng City, Jilin Province. Discovered in 1957, the Houtao site represents cultural remains from the Neolithic period. From July 2011 to October 2015, excavations were led by the Jilin Provincial Bureau of Cultural Relics and the School of Archaeology at Jilin University. The site can be divided into southern and northern areas by a large east-west trench in the middle of the site. Surface investigations show that relics from the Liao and Jin periods are predominantly found in the northern part of the northern area. In contrast, the central and southern parts of the northern area, as well as the southern area, are primarily characterized by Neolithic and Bronze Age remains. In this study, a total of 47 ancient individuals from the site were screened.

Balashan Late Neolithic burials in Liaoning, China

The Banlashan site is located in Chaoyang City, Liaoning Province. Banlashan is a stone mound burial site from the late Hongshan culture period, dating back more than 5,000 years. The site was discovered during the third national cultural relics census in 2009. In 2014, the Liaoning Provincial Institute of Cultural Relics and Archaeology, in conjunction with the Longcheng District Museum of Chaoyang City, jointly conducted excavations at the site. In this study, a total of 22 ancient individuals from the site were screened.

Lajia Early Bronze age burials in Qinghai, China

The Lajia site, located in Haidong, Qinghai Province, represents a major prehistoric settlement in the upper Yellow River region, predominantly associated with the Qijia culture. Discovered in 1981, the site underwent extensive archaeological excavations from 1999 to 2007, revealing significant Qijia cultural remains such as settlement dwellings, high-ranking tombs, defensive trenches, plazas, and altars, leading to numerous groundbreaking discoveries. Between 2013 and 2019, the site entered its second phase of excavation. Principal institutions involved in the excavation efforts include the Institute of Archaeology of the Chinese Academy of Social Sciences, the Qinghai Provincial Institute of Cultural Relics and Archaeology, the Minhe County Museum, the School of History and Culture at Sichuan University, the Chengdu Institute of Cultural Relics and Archaeology, and the Lajia Site Museum. In this study, a total of 38 ancient individuals from the site were screened.

Xiaohe Early Bronze age burials in Xinjiang, China

The Xiaohe site is located downstream of the Peacock River in the Lop Nur region. The lower layer of the Xiaohe cemetery dates back to 4000 years ago, while the upper layer dates to around 3500

years ago; both layers belong to the same cultural type. The Xiaohe cemetery was first discovered in 1900. Between 1927 and 1934, joint expeditions were conducted in the northwest region by Chinese and Swedish teams. From 2002 to 2005, the Xinjiang Institute of Archaeological Research conducted comprehensive excavations of the cemetery, uncovering 167 tombs and collecting over 30 well-preserved ancient mummies. In this study, a total of 45 ancient individuals from the site were screened.

Dingshishan Late Neolithic burials in Guangxi, China

The Dingshishan site, located in the Yongning District of the Guangxi Zhuang Autonomous Region, is an archaeological site from the late Neolithic period dating back approximately 5000 years. In 1994, the Yongning County Cultural Management Office conducted an initial survey of the site. In the autumn of 1996, a joint team from the Archaeological Institute of the Chinese Academy of Social Sciences in Guangxi, the Cultural Work Team of the Guangxi Zhuang Autonomous Region, and the Nanning City Museum revisited the site for further investigation. Between 1997 and 1999, four excavation campaigns were carried out at the site. Of the over 400 ancient human remains unearthed at the Dingshishan site to date, one-third were found to have been subjected to dismemberment burial. In this study, a total of 25 ancient individuals from the site were screened.

Dongsheng Early Iron Age and Historical period burials in Heilongjiang, China

The Dongsheng archaeological site is located in Hegang City, Heilongjiang Province. Its early cultural artifacts are from the Iron Age, while the later ones belong to the Ming and Qing dynasties. In the spring of 1991, the Middle Heilongjiang Cultural Relics Survey Team's Suibin and Luobei divisions discovered an ancient tomb complex in Suibin. In October of the same year, the Heilongjiang Provincial Institute of Cultural Relics and Archaeology dispatched a team to excavate 14 of these tombs. The historical artifacts unearthed from these graves confirmed that individuals from the Ming and Qing dynasties were the "Wudihaha" people, who are the ancestors of the modern-day Hezhen ethnic group, indicating that they once lived in this area. In this study, a total of 33 ancient individuals from the site were screened.

Xiguan Early Iron Age burials in Shanxi, China

The Xiguan archaeological site is located in Changzhi City, Shanxi Province. In 2005, it was discovered that the tombs at this site had been looted. Consequently, the Shanxi Provincial Institute of Archaeology dispatched archaeologists to investigate and excavate. After a year of archaeological work, a total of 92 tombs were identified in the Xiguan cemetery, of which 10 were excavated. The time span of these 10 tombs is not vast, all dating to the early Spring and Autumn period. In this study, a total of 23 ancient individuals from the site were screened.

Huangguxinle Neolithic burials in Liaoning, China

The Huangguxinle site, located in Shenyang, Liaoning Province, is a Neolithic matriarchal clan settlement site. The Shenyang Institute of Cultural Relics and Archaeology conducted six excavations on the site between 1973 and 2014, uncovering 44 architectural remains from the lower level of the Xinle culture. In this study, a total of 15 ancient individuals from the site were screened.

Dacaozi Historical burials in Qinghai, China

The Dacaozi site is located in Haidong City, Qinghai Province. It was discovered during the third national cultural relics survey in 2009 and was named the Dacaozi Han Tomb Cluster. Starting in 2014, the Qinghai Provincial Archaeological Team initiated rescue excavations at the Dacaozi Han Tomb Cluster. Based on the tomb structures and unearthed artifacts, the archaeologists determined that the Dacaozi Han Tomb Cluster roughly spans the periods of the Eastern Han, Wei, Jin, the Sixteen Kingdoms, and the Song-Yuan dynasties. In this study, a total of 29 ancient individuals from the site were screened.

Erdaojingzi Early Bronze age burials in Inner Mongolia, China

The Erdaojingzi site is located in Chifeng City, Inner Mongolia Autonomous Region. It belongs to the lower stratum of the Xiajiadian culture, dating back roughly 4,000 years. At the beginning of 2009, the Inner Mongolia Institute of Cultural Relics and Archaeology conducted a year-long rescue excavation at the site. During this process, they uncovered 305 relics including city walls, moats, courtyards, houses, roads, cellars, ash pits, and tombs. In this study, a total of 18 ancient individuals from the site were screened.

Haminmangha Neolithic burials in Inner Mongolia, China

The Haminmangha archaeological site, a prehistoric settlement, is located in Tongliao City, Inner Mongolia. From May to September 2010, a joint archaeological team comprised of the Inner Mongolia Institute of Cultural Relics and Archaeology and the Cultural Relics Administration Office of Kezuozhong Banner in Tongliao City conducted a cultural relics survey. During this period, they discovered that the Haminmangha site was being illicitly excavated. Subsequently, the Inner Mongolia Institute of Cultural Relics and Archaeology, in collaboration with the Frontier Archaeology Research Center of Jilin University, carried out extensive archaeological excavations at the site. Over two years of excavation, the team unearthed 43 house foundations, 6 tombs, 33 ash pits, and a single moat. The third excavation of the ancient site concluded comprehensively on July 25, 2012. The Haminmangha site dates back to the Neolithic period, approximately between 6,000 and 5,000 years ago. In this study, a total of 14 ancient individuals from the site were screened.

Miaozigou Neolithic burials in Inner Mongolia, China

The Miaozigou site is located in the Ulanqab city of the Inner Mongolia Autonomous Region and belongs to the late Neolithic period. In October 1985, this primitive village site, which dates back about 5,500 years, was discovered. Between May and September 1987, the Inner Mongolia Institute of Cultural Relics and Archaeology, together with the Cultural Relics Management Office of the Qarqin Front Banner, carried out extensive excavations. At the Miaozigou site, a total of 51 house foundations, 43 tombs, and 132 storage pits (ash pits) were uncovered. In this study, a total of 9 ancient individuals from the site were screened.

Mogushan Early Iron Age burials in Inner Mongolia, China

The Mogushan site, located in Hulunbuir City of the Inner Mongolia Autonomous Region, is a cultural site from the Paleolithic period. In 1903, the area near the Mogushan site was excavated. During the excavation, a large number of ancient biological and human fossils were discovered, along with many small stone tools and pottery shards. The Mogushan site was identified in 1980. In 1990, a detailed survey and trial excavation were conducted. In this study, a total of 6 ancient

individuals from the site were screened.

Pingliangtai Late Neolithic burials in Henan, China

The Pingliangtai site is located in Zhoukou City, Henan Province. The site was first discovered in 1979. With a history of over 4,600 years, it represents an urban site from the late Neolithic Longshan Culture in China. In this study, a total of 19 ancient individuals from the site were screened.

Gumugou Early Bronze age burials in Xinjiang, China

The Gumugou site is located downstream of the Peacock River in Lop Nur, Xinjiang. It was discovered in 1979 by the Xinjiang Institute of Cultural Relics and Archaeology, with 42 tombs excavated and documented. The site represents a communal burial ground from primitive society. Based on dating methods, these remains are attributed to the indigenous cultures of the Lop Nur region from approximately 3,800 years ago. In this study, a total of 14 ancient individuals from the site were screened.

Haojiatai Early Bronze age burials in Henan, China

The Haojiatai site is located in Luohe City, Henan Province. It was first discovered in May 1986. From June 1986 to May 1987, the Henan Institute of Cultural Relics and Archaeology, together with the Luohe City Cultural Bureau and the Shaocheng County Cultural Bureau, jointly conducted archaeological excavations at the site. They uncovered 14 house foundations from the Longshan and Erlitou cultures, over 310 ash pits, and more than 90 tombs. The site provides insights into the historical and cultural developments of the region east of the Song Mountains in the Huang-Huai River Basin from 4,000 to 5,000 years ago. In this study, a total of 12 ancient individuals from the site were screened.

Section 3: Figs. S1 to S10

Fig. S1. Geographical distribution, dating, and genotype of our ancient samples. As indicated in the legend, the different colors represent the different HBV genotypes. The sample name and period for the newly reconstructed ancient HBV genomes are consistent with the color of the genotype. B* represents the genotype B without a recombination event with genotype C. B# represents the recombinant.

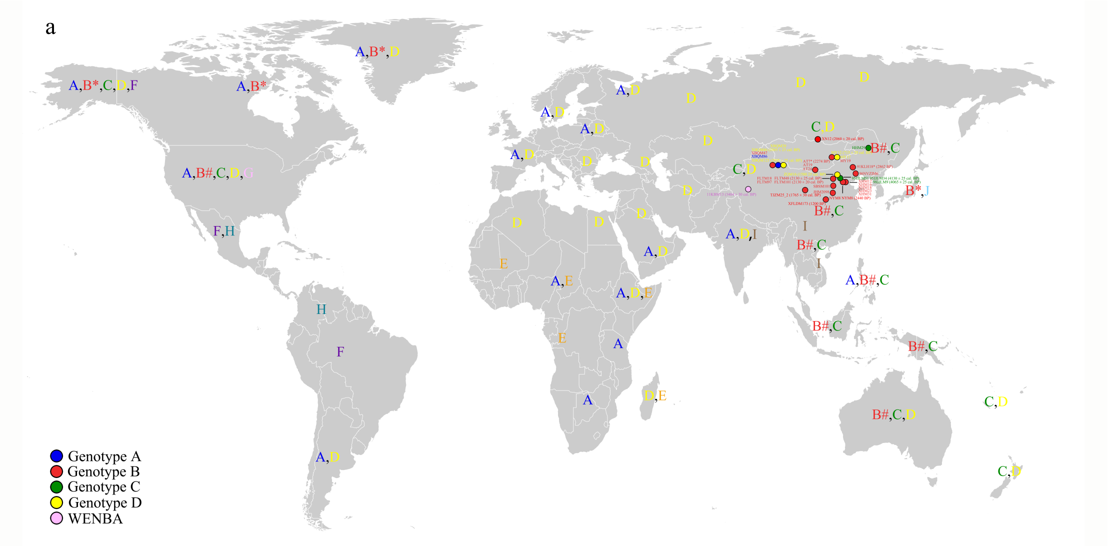

Fig. S2. Damage pattern of all the HBV positive samples to HBV and human, calculated by mapDamage v.2.0.9-dirty[9]. Each line represents a sample. Blue lines represent to human and orange lines represent to HBV.

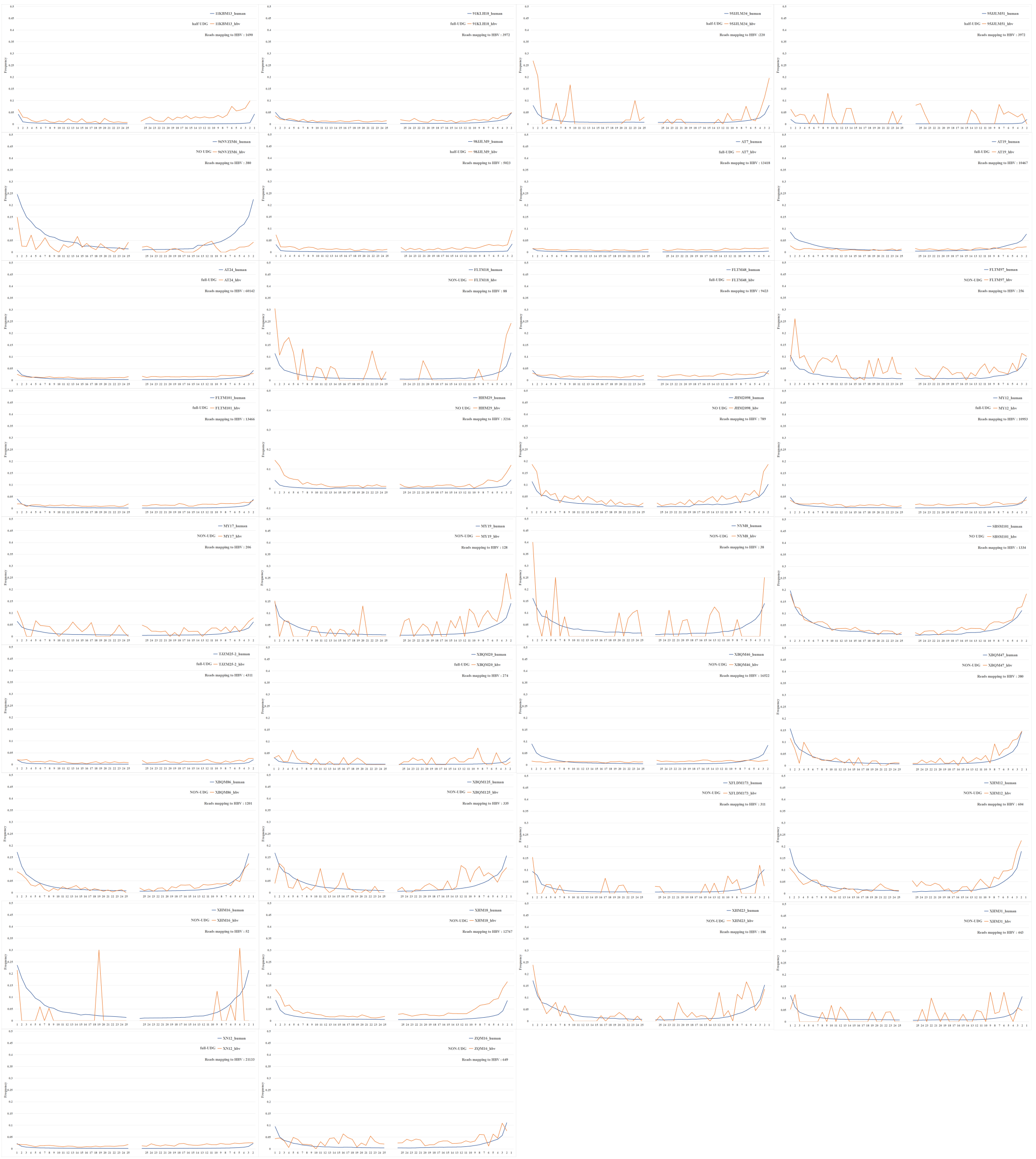

Fig.S3. Maximum likelihood tree built with RAxML[10]**.** Highlighted in different colors are the branches of the new ancient HBV sequences being: blue - genotype A, red - genotype B, orange - genotype C, green - genotype D, and purple - WENBA. The bootstrap values are displayed for each of the nodes. a The maximum likelihood tree containing individuals with mixed infections. b The maximum likelihood tree without mixed infections individuals. c The maximum likelihood tree including DA45, with DA45 and AT19 highlighted in red, and a bootstrap value of 100. d The maximum likelihood trees constructed individually for each genotype.

a

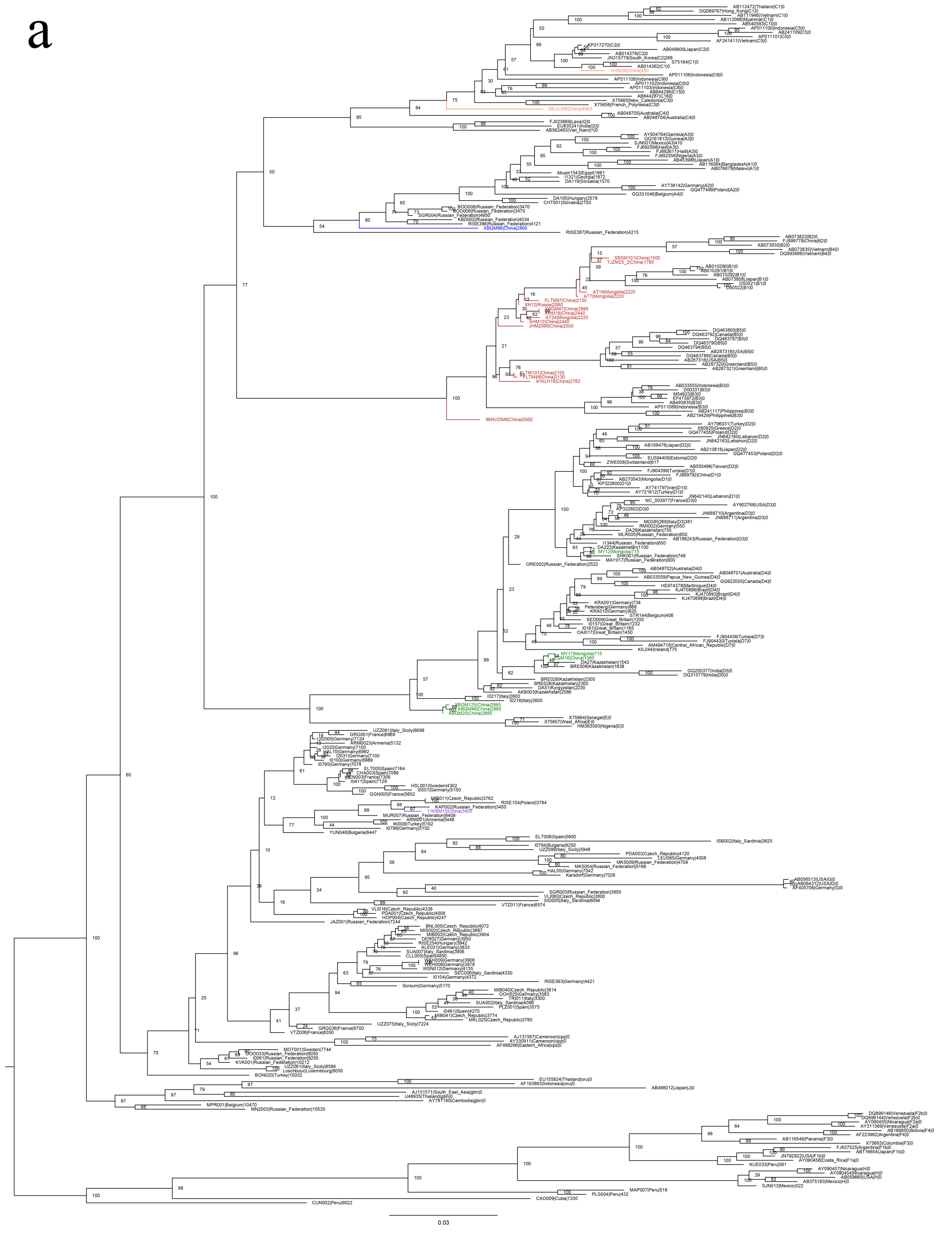

b

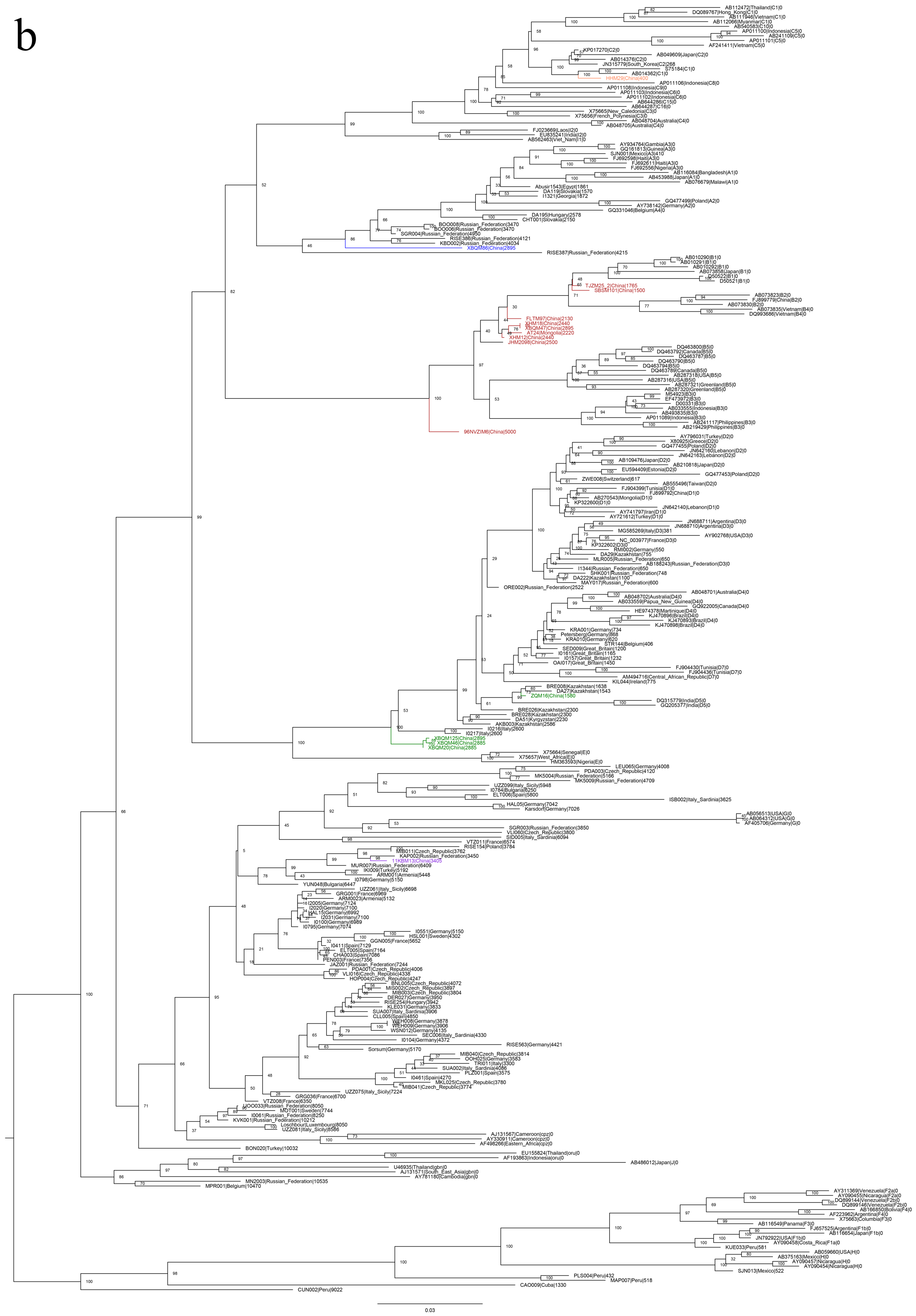

c

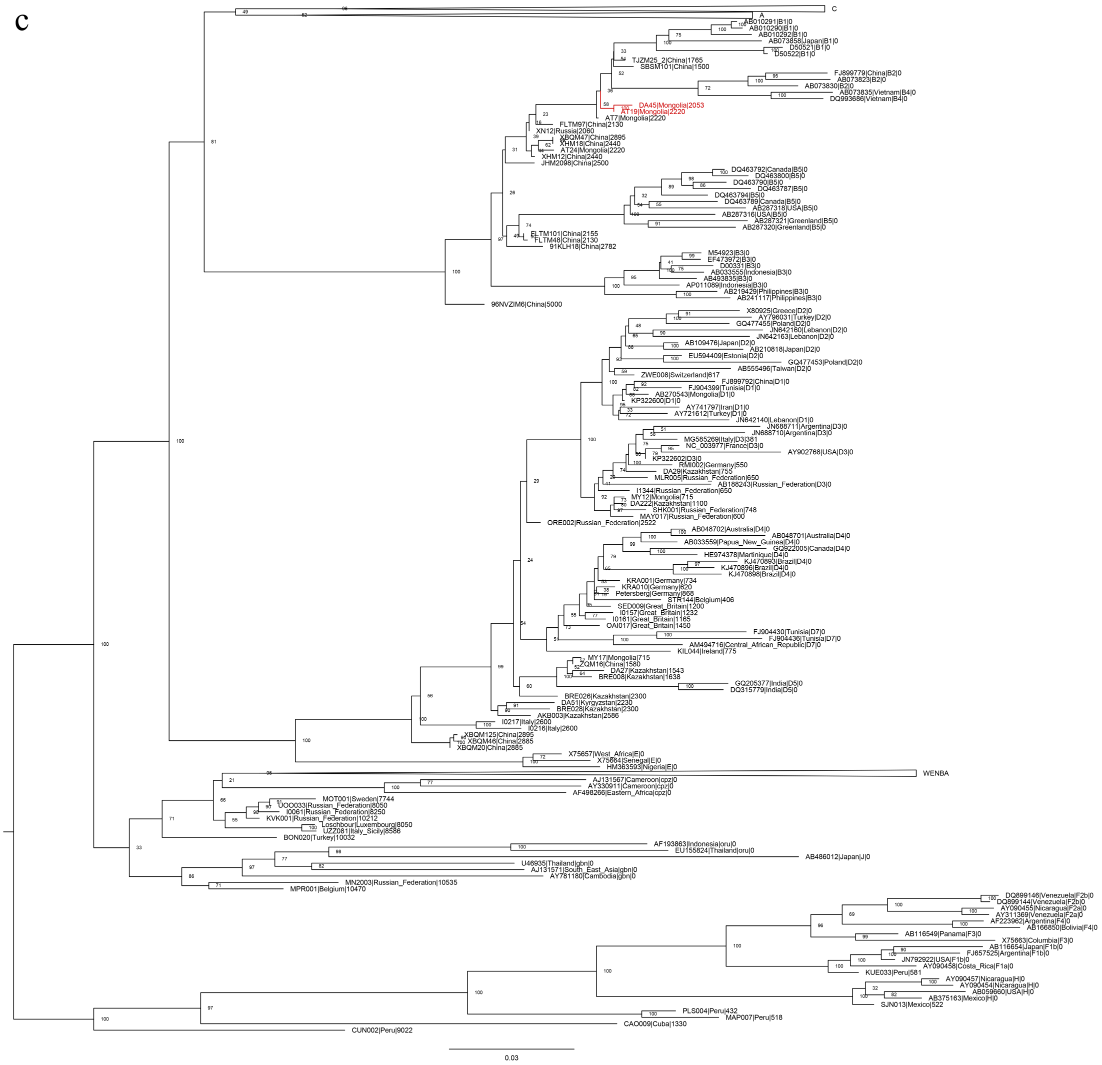
C
A
AB010291|B1|0
AB010290|B1|0
AB010292|B1|0
AB073858|Japan|B1|0
D50521|B1|0
D50522|B1|0
TJZM25_2|China|1765
SBSM101|China|1500
FJ899779|China|B2|0
AB073823|B2|0
AB073830|B2|0
AB073835|Vietnam|B4|0
DQ993686|Vietnam|B4|0
DA45|Mongolia|2053
AT19|Mongolia|2220
AT7|Mongolia|2220
FLTM97|China|2130
XN12|Russia|2060
XBQM47|China|2895
XHM18|China|2440
AT24|Mongolia|2220
XHM12|China|2440
JHM2098|China|2500
DQ463792|Canada|B5|0
DQ463800|B5|0
DQ463790|B5|0
DQ463787|B5|0
DQ463794|B5|0
DQ463789|Canada|B5|0
AB287318|USA|B5|0
AB287316|USA|B5|0
AB287321|Greenland|B5|0
AB287320|Greenland|B5|0
FLTM101|China|2155
FLTM48|China|2130
91KLH18|China|2782
M54923|B3|0
EF473972|B3|0
D00331|B3|0
AB033555|Indonesia|B3|0
AB493835|B3|0
AP011089|Indonesia|B3|0
AB219429|Philippines|B3|0
AB241117|Philippines|B3|0
96NVZIM6|China|5000
X80925|Greece|D2|0
AY796031|Turkey|D2|0
GQ477455|Poland|D2|0
JN642160|Lebanon|D2|0
JN642163|Lebanon|D2|0
AB109476|Japan|D2|0
AB210818|Japan|D2|0
EU594409|Estonia|D2|0
GQ477453|Poland|D2|0
AB555496|Taiwan|D2|0
ZWE008|Switzerland|617
FJ899792|China|D1|0
FJ904399|Tunisia|D1|0
AB270543|Mongolia|D1|0
KP322600|D1|0
AY741797|Iran|D1|0
AY721612|Turkey|D1|0
JN642140|Lebanon|D1|0
JN688711|Argentina|D3|0
JN688710|Argentina|D3|0
MG585269|Italy|D3|381
NC_003977|France|D3|0
AY902768|USA|D3|0
KP322602|D3|0
RMI002|Germany|550
DA29|Kazakhstan|755
MLR005|Russian_Federation|650
AB188243|Russian_Federation|D3|0
I1344|Russian_Federation|650
MY12|Mongolia|715
DA222|Kazakhstan|1100
SHK001|Russian_Federation|748
MAY017|Russian_Federation|600
ORE002|Russian_Federation|2522
AB048702|Australia|D4|0
AB048701|Australia|D4|0
AB033559|Papua_New_Guinea|D4|0
GQ922005|Canada|D4|0
HE974378|Martinique|D4|0
KJ470893|Brazil|D4|0
KJ470896|Brazil|D4|0
KJ470898|Brazil|D4|0
KRA001|Germany|734
KRA010|Germany|620
Petersberg|Germany|868
STR144|Belgium|406
SED009|Great_Britain|1200
I0157|Great_Britain|1232
I0161|Great_Britain|1165
OAI017|Great_Britain|1450
FJ904430|Tunisia|D7|0
FJ904436|Tunisia|D7|0
AM494716|Central_African_Republic|D7|0
KIL044|Ireland|775
MY17|Mongolia|715
ZQM16|China|1580
DA27|Kazakhstan|1543
BRE008|Kazakhstan|1638
GQ205377|India|D5|0
DQ315779|India|D5|0
BRE026|Kazakhstan|2300
DA51|Kyrgyzstan|2230
BRE028|Kazakhstan|2300
AKB003|Kazakhstan|2586
I0217|Italy|2600
I0216|Italy|2600
XBQM125|China|2895
XBQM46|China|2885
XBQM20|China|2885
X75657|West_Africa|E|0
X75664|Senegal|E|0
HM363593|Nigeria|E|0
WENBA
AJ131567|Cameroon|cpz|0
AY330911|Cameroon|cpz|0
AF498266|Eastern_Africa|cpz|0
MOT001|Sweden|7744
UOO033|Russian_Federation|8050
I0061|Russian_Federation|8250
KVK001|Russian_Federation|10212
Loschbour|Luxembourg|8050
UZZ081|Italy_Sicily|8586
BON020|Turkey|10032
AF193863|Indonesia|oru|0
EU155824|Thailand|oru|0
AB486012|Japan|J|0
U46935|Thailand|gbn|0
AJ131571|South_East_Asia|gbn|0
AY781180|Cambodia|gbn|0
MN2003|Russian_Federation|10535
MPR001|Belgium|10470
DQ899146|Venezuela|F2b|0
DQ899144|Venezuela|F2b|0
AY090455|Nicaragua|F2a|0
AY311369|Venezuela|F2a|0
AF223962|Argentina|F4|0
AB166850|Bolivia|F4|0
AB116549|Panama|F3|0
X75663|Columbia|F3|0
AB116654|Japan|F1b|0
FJ657525|Argentina|F1b|0
JN792922|USA|F1b|0
AY090458|Costa_Rica|F1a|0
KUE033|Peru|581
AY090457|Nicaragua|H|0
AY090454|Nicaragua|H|0
AB059660|USA|H|0
AB375163|Mexico|H|0
SJN013|Mexico|522
PLS004|Peru|432
MAP007|Peru|518
CAO009|Cuba|1330
CUN002|Peru|9022
0.03

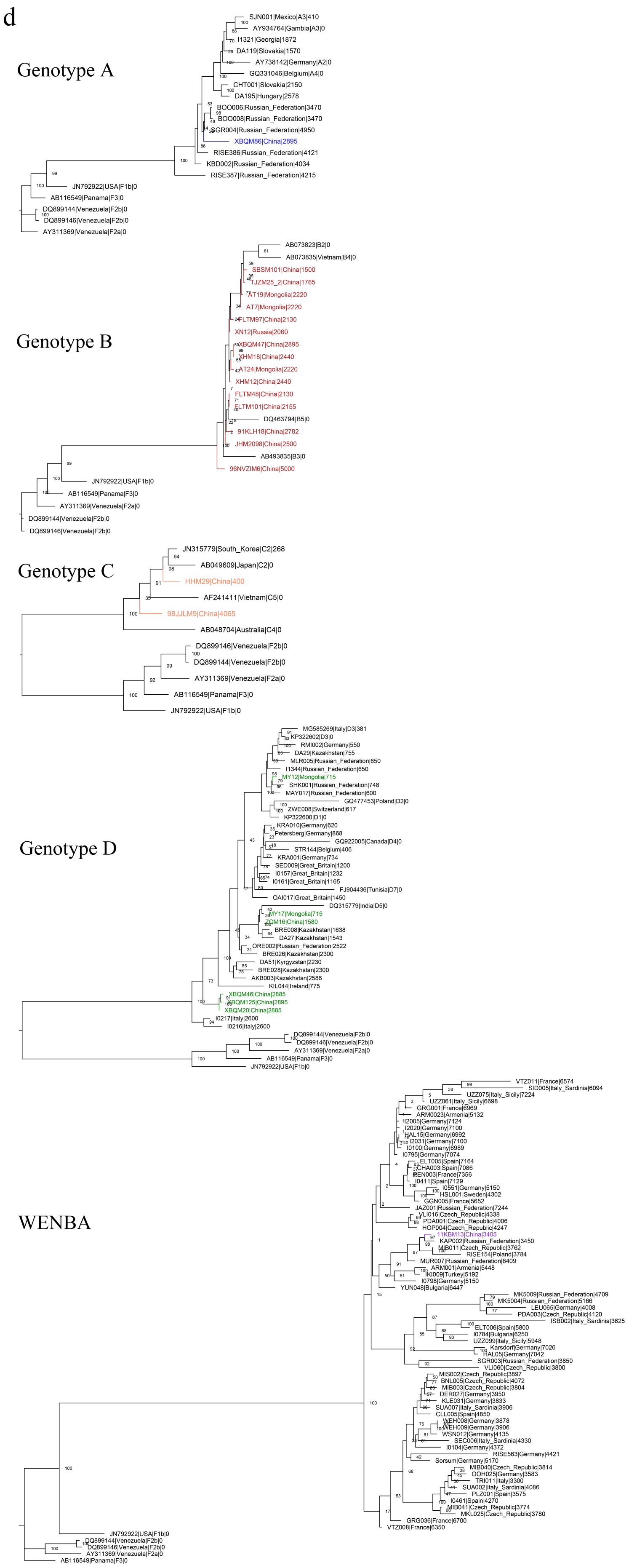
d
Genotype A
SJN001|Mexico|A3|410
AY934764|Gambia|A3|0
I1321|Georgia|1872
DA119|Slovakia|1570
AY738142|Germany|A2|0
GQ331046|Belgium|A4|0
CHT001|Slovakia|2150
DA195|Hungary|2578
BOO006|Russian_Federation|3470
BOO008|Russian_Federation|3470
SGR004|Russian_Federation|4950
XBQM86|China|2895
RISE386|Russian_Federation|4121
KBD002|Russian_Federation|4034
RISE387|Russian_Federation|4215
JN792922|USA|F1b|0
AB116549|Panama|F3|0
DQ899144|Venezuela|F2b|0
DQ899146|Venezuela|F2b|0
AY311369|Venezuela|F2a|0
Genotype B
AB073823|B2|0
AB073835|Vietnam|B4|0
SBSM101|China|1500
TJZM25_2|China|1765
AT19|Mongolia|2220
AT7|Mongolia|2220
FLTM97|China|2130
XN12|Russia|2060
XBQM47|China|2895
XHM18|China|2440
AT24|Mongolia|2220
XHM12|China|2440
FLTM48|China|2130
FLTM101|China|2155
DQ463794|B5|0
91KLH18|China|2782
JHM2098|China|2500
AB493835|B3|0
96NVZIM6|China|5000
JN792922|USA|F1b|0
AB116549|Panama|F3|0
AY311369|Venezuela|F2a|0
DQ899144|Venezuela|F2b|0
DQ899146|Venezuela|F2b|0
Genotype C
JN315779|South_Korea|C2|268
AB049609|Japan|C2|0
HHM29|China|400
AF241411|Vietnam|C5|0
98JJLM9|China|4065
AB048704|Australia|C4|0
DQ899146|Venezuela|F2b|0
DQ899144|Venezuela|F2b|0
AY311369|Venezuela|F2a|0
AB116549|Panama|F3|0
JN792922|USA|F1b|0
Genotype D
MG585269|Italy|D3|381
KP322602|D3|0
RMI002|Germany|550
DA29|Kazakhstan|755
MLR005|Russian_Federation|650
I1344|Russian_Federation|650
MY12|Mongolia|715
SHK001|Russian_Federation|748
MAY017|Russian_Federation|600
GQ477453|Poland|D2|0
ZWE008|Switzerland|617
KP322600|D1|0
KRA010|Germany|620
Petersberg|Germany|868
GQ922005|Canada|D4|0
STR144|Belgium|406
KRA001|Germany|734
SED009|Great_Britain|1200
I0157|Great_Britain|1232
I0161|Great_Britain|1165
FJ904436|Tunisia|D7|0
OAI017|Great_Britain|1450
DQ315779|India|D5|0
MY17|Mongolia|715
ZQM16|China|1580
BRE008|Kazakhstan|1638
DA27|Kazakhstan|1543
ORE002|Russian_Federation|2522
BRE026|Kazakhstan|2300
DA51|Kyrgyzstan|2230
BRE028|Kazakhstan|2300
AKB003|Kazakhstan|2586
KIL044|Ireland|775
XBQM46|China|2885
XBQM125|China|2895
XBQM20|China|2885
I0217|Italy|2600
I0216|Italy|2600
DQ899144|Venezuela|F2b|0
DQ899146|Venezuela|F2b|0
AY311369|Venezuela|F2a|0
AB116549|Panama|F3|0
JN792922|USA|F1b|0
WENBA
VTZ011|France|6574
SID005|Italy_Sardinia|6094
UZZ075|Italy_Sicily|7224
UZZ061|Italy_Sicily|6698
GRG001|France|6969
ARM0023|Armenia|5132
I2005|Germany|7124
I2020|Germany|7100
HAL15|Germany|6992
I2031|Germany|7100
I0100|Germany|6989
I0795|Germany|7074
ELT005|Spain|7164
CHA003|Spain|7086
BEN003|France|7356
I0411|Spain|7129
I0551|Germany|5150
HSL001|Sweden|4302
GGN005|France|5652
JAZ001|Russian_Federation|7244
VLI016|Czech_Republic|4338
PDA001|Czech_Republic|4006
HOP004|Czech_Republic|4247
11KBM13|China|3405
KAP002|Russian_Federation|3450
MIB011|Czech_Republic|3762
RISE154|Poland|3784
MUR007|Russian_Federation|6409
ARM001|Armenia|5448
IKI009|Turkey|5192
I0798|Germany|5150
YUN048|Bulgaria|6447
MK5009|Russian_Federation|4709
MK5004|Russian_Federation|5166
LEU065|Germany|4008
PDA003|Czech_Republic|4120
ISB002|Italy_Sardinia|3625
ELT006|Spain|5800
I0784|Bulgaria|6250
UZZ099|Italy_Sicily|5948
Karsdorf|Germany|7026
HAL05|Germany|7042
SGR003|Russian_Federation|3850
VLI060|Czech_Republic|3800
MIS002|Czech_Republic|3897
BNL005|Czech_Republic|4072
MIB003|Czech_Republic|3804
DER027|Germany|3950
KLE031|Germany|3833
SUA007|Italy_Sardinia|3906
CLL005|Spain|4850
WEH008|Germany|3878
WEH009|Germany|3906
WSN012|Germany|4135
SEC006|Italy_Sardinia|4330
I0104|Germany|4372
RISE563|Germany|4421
Sorsum|Germany|5170
MIB040|Czech_Republic|3814
OOH025|Germany|3583
TRI011|Italy|3300
SUA002|Italy_Sardinia|4086
PLZ001|Spain|3575
I0461|Spain|4270
MIB041|Czech_Republic|3774
MKL025|Czech_Republic|3780
GRG036|France|6700
VTZ008|France|6350
JN792922|USA|F1b|0
DQ899144|Venezuela|F2b|0
DQ899146|Venezuela|F2b|0
AY311369|Venezuela|F2a|0
AB116549|Panama|F3|0

Fig.S4. Regression of root-to-tip genetic distances against tip dates. R squared and correlation coefficient are displayed in the upper-left corner calculated with Tempest (v.1.5.3).[11]

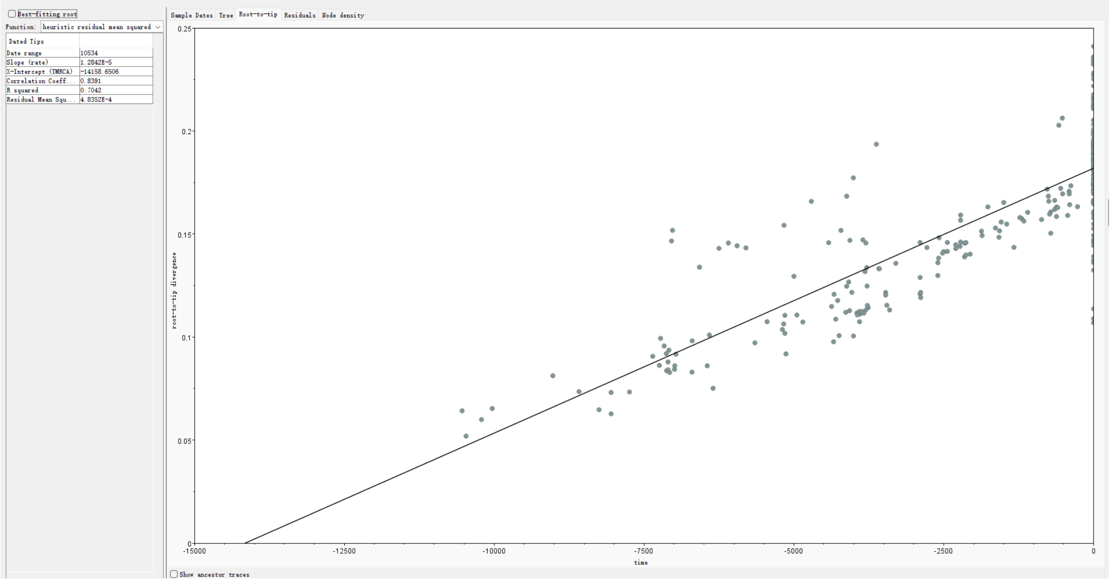

Fig.S5 MCC time-calibrated phylogenetic tree of modern and ancient HBV with WENBA uncollapsed. The lineages in which our ancient sequences are located are highlighted with different colors: blue represents genotype A genomes, red genotype B, orange genotype C, green genotype D, and purple WENBA lineage. The branches and names of the ancient sequences are also highlighted with the indicated color. a MCC time-calibrated phylogenetic tree without mixed infections individuals. b MCC time-calibrated phylogenetic tree display the most recent common ancestor. The blue horizontal bars represent the 95% confidence interval of tMCRA and the number of 95% confidence interval is next to the blue horizontal bars.

a

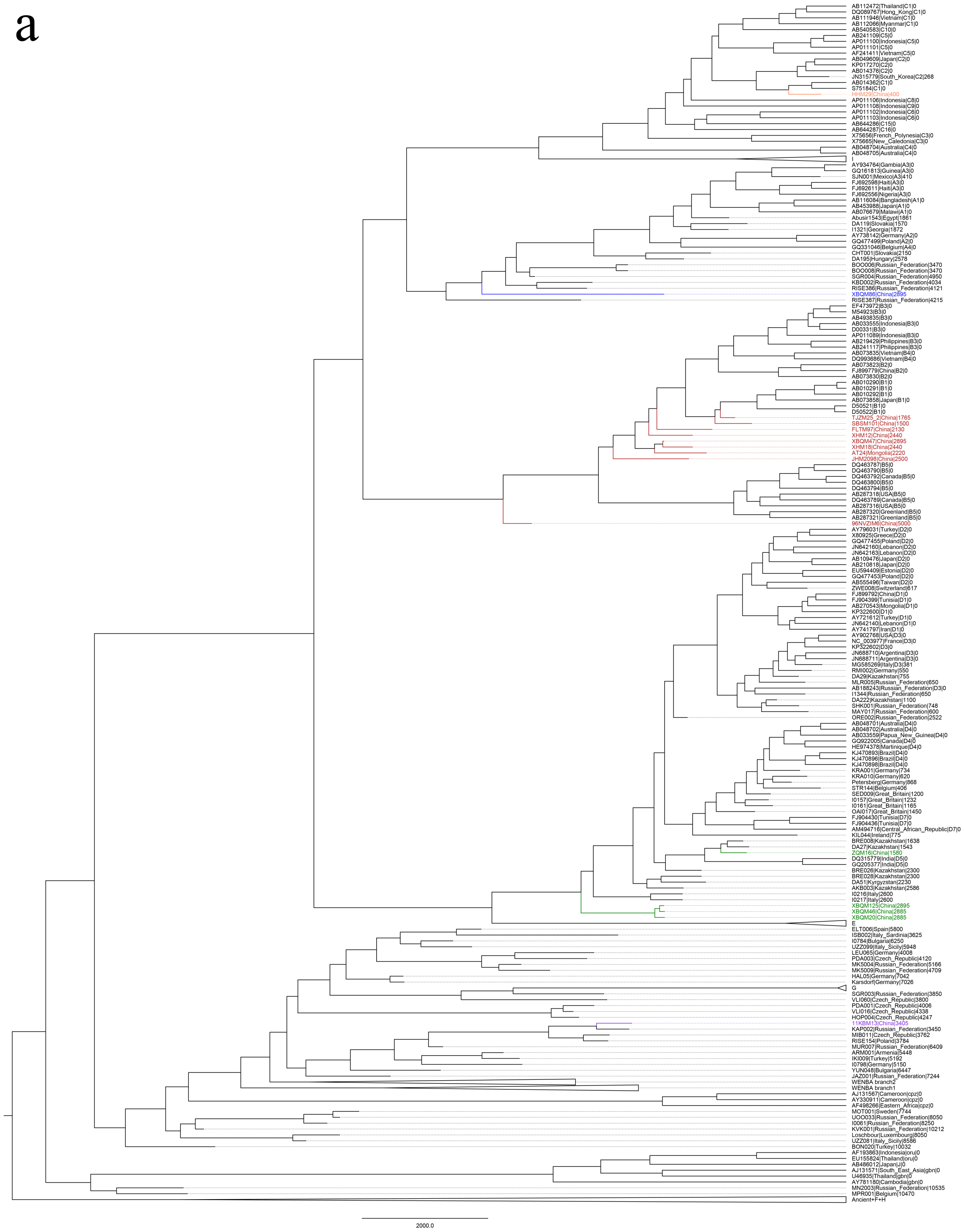

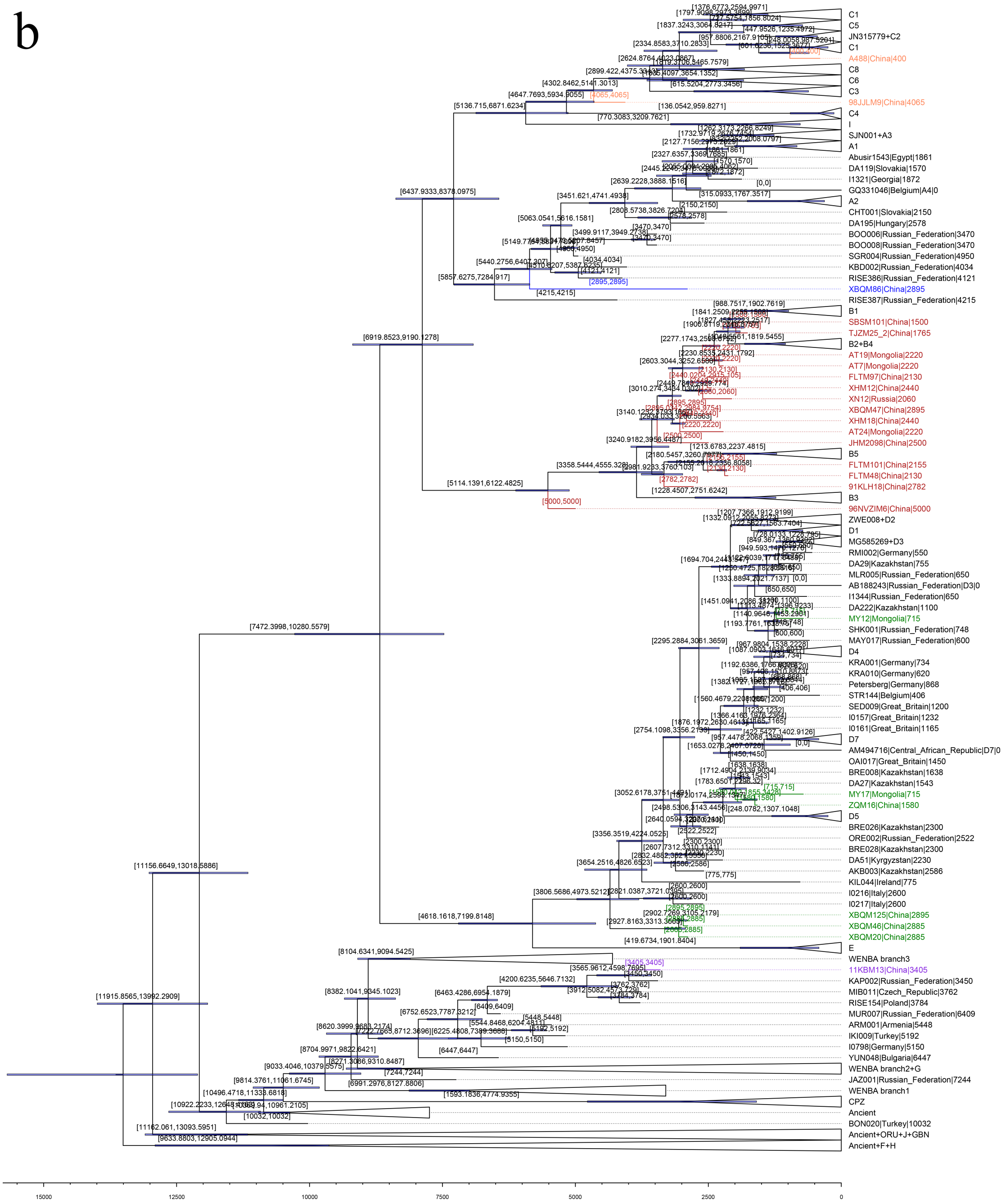
b
C1
C5
JN315779+C2
C1
A488|China|400
C8
C6
C3
98JJLM9|China|4065
C4
I
SJN001+A3
A1
Abusir1543|Egypt|1861
DA119|Slovakia|1570
I1321|Georgia|1872
GQ331046|Belgium|A4|0
A2
CHT001|Slovakia|2150
DA195|Hungary|2578
BOO006|Russian_Federation|3470
BOO008|Russian_Federation|3470
SGR004|Russian_Federation|4950
KBD002|Russian_Federation|4034
RISE386|Russian_Federation|4121
XBQM86|China|2895
RISE387|Russian_Federation|4215
B1
SBSM101|China|1500
TJZM25_2|China|1765
B2+B4
AT19|Mongolia|2220
AT7|Mongolia|2220
FLTM97|China|2130
XHM12|China|2440
XN12|Russia|2060
XBQM47|China|2895
XHM18|China|2440
AT24|Mongolia|2220
JHM2098|China|2500
B5
FLTM101|China|2155
FLTM48|China|2130
91KLH18|China|2782
B3
96NVZIM6|China|5000
ZWE008+D2
D1
MG585269+D3
RMI002|Germany|550
DA29|Kazakhstan|755
MLR005|Russian_Federation|650
AB188243|Russian_Federation|D3|0
I1344|Russian_Federation|650
DA222|Kazakhstan|1100
MY12|Mongolia|715
SHK001|Russian_Federation|748
MAY017|Russian_Federation|600
D4
KRA001|Germany|734
KRA010|Germany|620
Petersberg|Germany|868
STR144|Belgium|406
SED009|Great_Britain|1200
I0157|Great_Britain|1232
I0161|Great_Britain|1165
D7
AM494716|Central_African_Republic|D7|0
OAI017|Great_Britain|1450
BRE008|Kazakhstan|1638
DA27|Kazakhstan|1543
MY17|Mongolia|715
ZQM16|China|1580
D5
BRE026|Kazakhstan|2300
ORE002|Russian_Federation|2522
BRE028|Kazakhstan|2300
DA51|Kyrgyzstan|2230
AKB003|Kazakhstan|2586
KIL044|Ireland|775
I0216|Italy|2600
I0217|Italy|2600
XBQM125|China|2895
XBQM46|China|2885
XBQM20|China|2885
E
WENBA branch3
11KBM13|China|3405
KAP002|Russian_Federation|3450
MIB011|Czech_Republic|3762
RISE154|Poland|3784
MUR007|Russian_Federation|6409
ARM001|Armenia|5448
IKI009|Turkey|5192
I0798|Germany|5150
YUN048|Bulgaria|6447
WENBA branch2+G
JAZ001|Russian_Federation|7244
WENBA branch1
CPZ
Ancient
BON020|Turkey|10032
Ancient+ORU+J+GBN
Ancient+F+H
15000
12500
10000
7500
5000
2500
0

Fig.S6. Unrooted phylogenetic network. a Unrooted tree display label name. b Unrooted tree without label name.

a

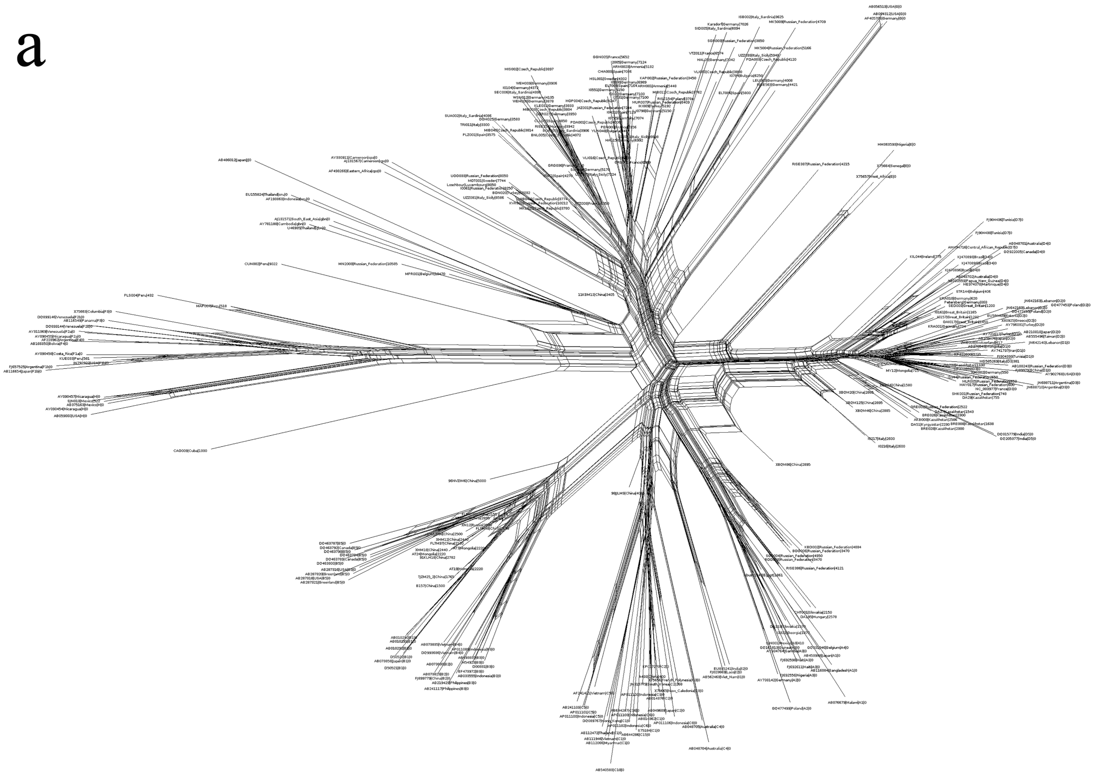

b

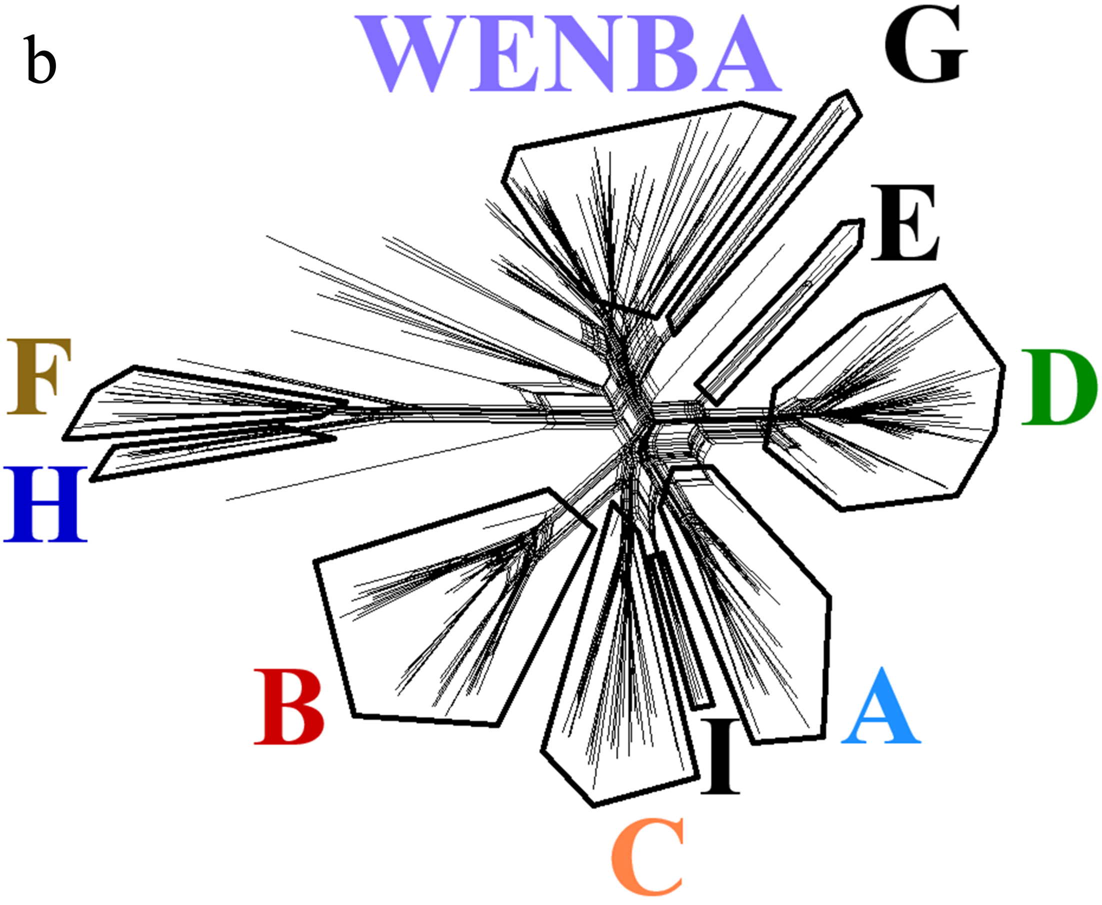

Fig.S7. Recombination analysis results using RDP5. a The red line represents the sequence identity between the major parent and recombinant. The green line represents the sequence identity between the minor parent and recombinant. b The blue line represents the sequence identity between the major parent and minor parent. The orange line represents the sequence identity between the major parent and recombinant. The gray line represents the sequence identity between the minor parent and recombinant.

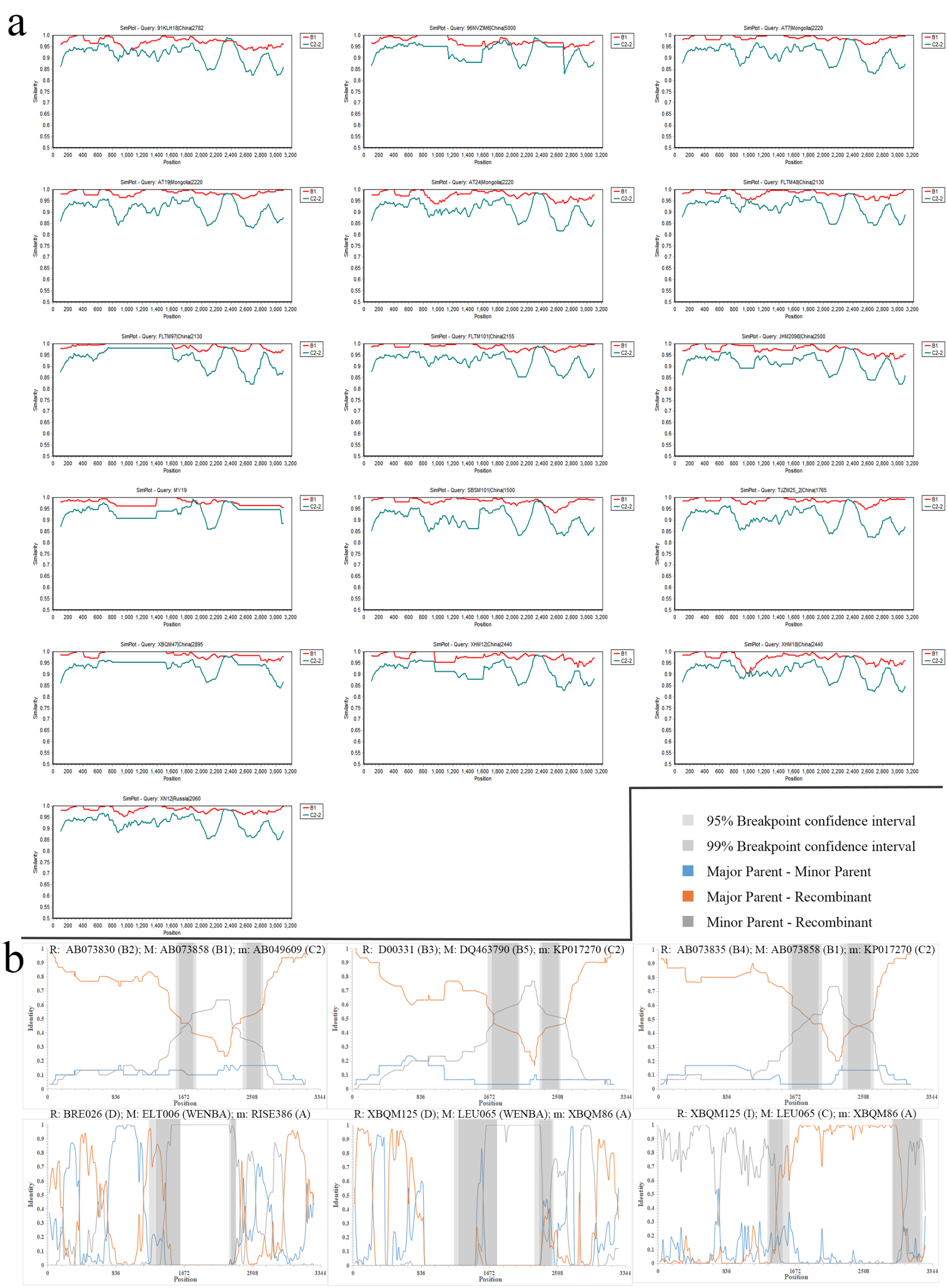

Fig.S8. PCA and Admixture results with each individual mark. a Principal component analysis of ancient individuals infected with HBV and present-day individuals. The first two principal components were constructed from 2077 present-day Eurasians; the ancient individuals are projected onto the first two PCs. Each individual were marked with a different shape. The Gray asterisk represents the present-day individuals used for calculating PCs. b ADMIXTURE results for the “1240k-Illumina” dataset at K = 9. c Here we show the population labels for the 2,077 Eurasian individuals used for calculating the PCs and plotted as a gray asterisk in Figure 4a. Each three-letter code in the plot represents a single individual. Population IDs matching the three-letter codes are listed at the bottom.

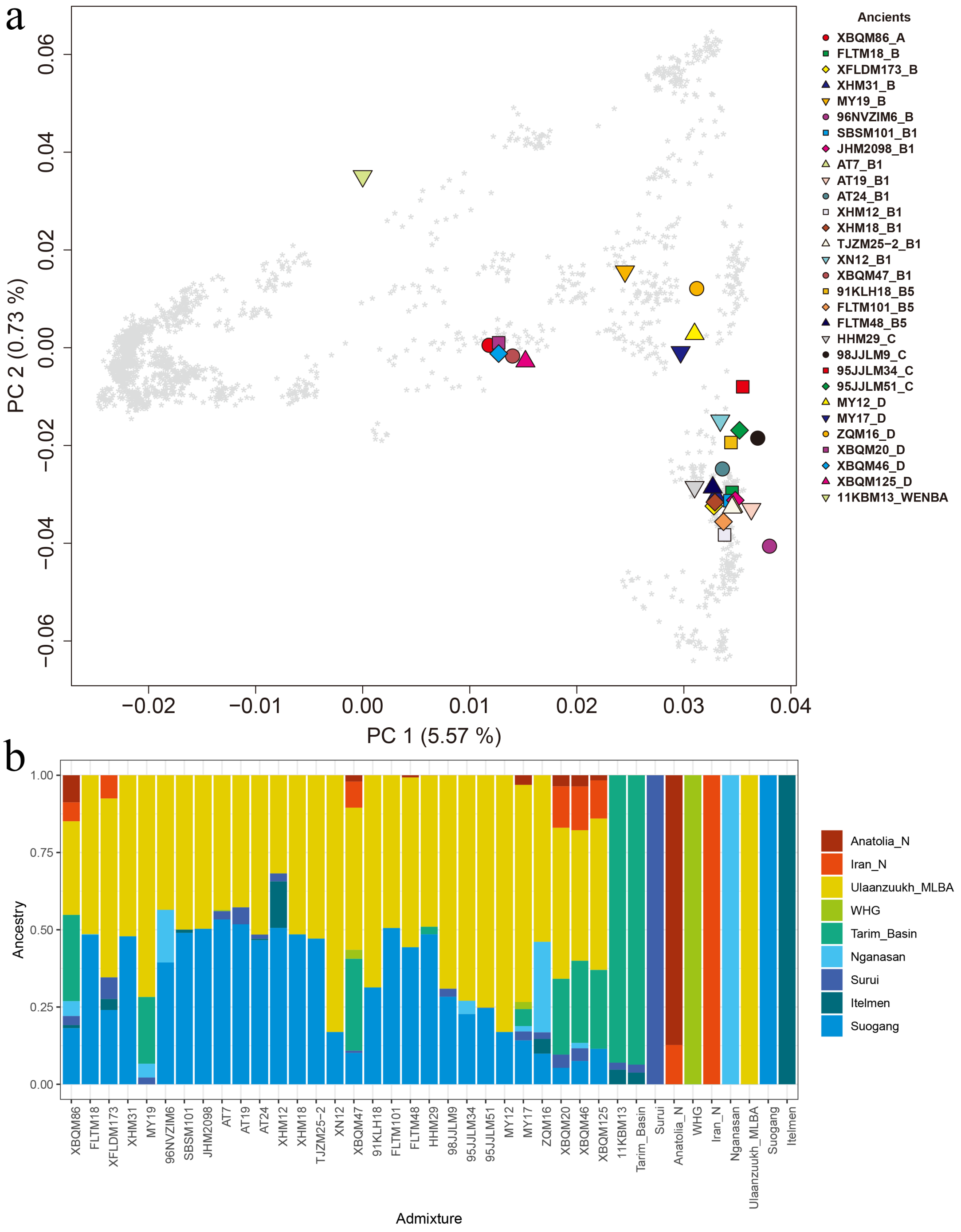

c

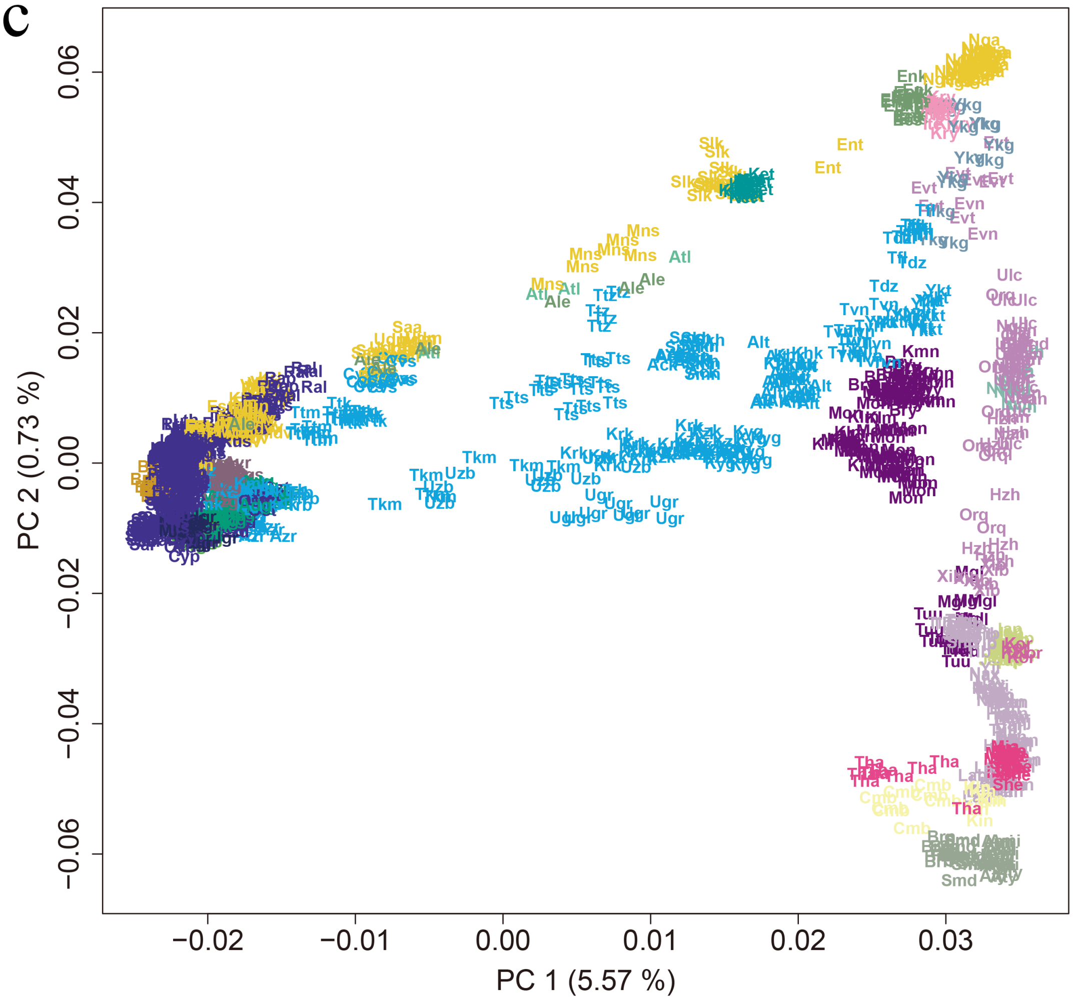

Abz Abazin	Ccs Circassian	Iri Irish	Krd Kurd	Rom Romanian	Ttz Tatar_Zabolotniye
Abk Abkhasian	Crt Croatian	Iru Irish_Ulster	Kyg Kyrgyz	Rus Russian	Tha Thai
Adg Adygei	Cyp Cypriot	Itn Italian_North	Lah Lahu	Rak Russian_Krasnoborsky	Tib Tibetan
Abn Albanian	Cze Czech	Its Italian_South	Lak Lak	Ral Russian_Leshukonsky	Tdz Todzin
Ale Aleut	Ent Enets	Ite Itelmen	Lzg Lezgin	Rap Russian_Pinezhsky	Tfl Tofalar
Atl Aleut_Tlingit	Eng English	Jap Japanese	Lth Lithuanian	Saa Saami	Tuu Tu
Alt Altaian	Ecs Eskimo_CS	Jas Jew_Ashkenazi	Mlt Maltese	Sar Sardinian	Tbl Tubalar
Ack Altaian_Chelkans	Enk Eskimo_NK	Jgr Jew_Georgian	Mns Mansi	Sct Scottish	Tuj Tujia
Ami Ami	Est Estonian	Kbd Kabardinian	Mia Miao	Slk Selkup	Tra Turkish
Arm Armenian	Evn Even	Ktg Kaitag	Mld Moldavian	Smd Semende	Trb Turkish_Balikesir
Ahm Armenian_Hemsheni	Evf Evenk_FarEast	Klm Kalmyk	Mon Mongol	She She	Tkm Turkmen
Aty Atayal	Evt Evenk_Transbaikal	Krc Karachai	Mgl Mongola	Shp Sherpa	Tvn Tuvinian
Avr Avar	Ezd Ezid	Krk Karakalpak	Mdv Mordovian	Stl Shetlandic	Udm Udmurt
Azr Azeri	Fin Finnish	Krl Karelian	Nan Nanai	Skh Shor_Khakassia	Ukr Ukrainian
Blk Balkar	Fre French	Kzk Kazakh	Nax Naxi	Smn Shor_Mountain	Ulc Ulchi
Bsc Bashkir	Ggz Gagauz	Ket Ket	Ngd Negidal	Scl Sicilian	Ugr Uygur
Bsq Basque	Grg Georgian	Khs Khakass	Nga Nganasan	Srb Sorb	Uzb Uzbek
Blr Belarusian	Ger German	Khk Khakass_Kachins	Nvh Nivh	Spa Spanish	Vep Veps
Bes Besermyan	Grk Greek	Kmn Khamnegan	Ng1 Nogai	Spn Spanish_North	Xib Xibo
Brn Borneo	Han Han	Kin Kinh	Nwg Norwegian	Tbs Tabasaran	Ykt Yakut
Blg Bulgarian	Hzh Hezhen	Kor Korean	Orc Orcadian	Tjl Tajik	Yii Yi
Bry Buryat	Hun Hungarian	Kry Koryak	Orq Oroqen	Ttk Tatar_Kazan	Ykg Yukagir
Cmb Cambodian	Ice Icelandic	Kbc Kubachinian	Ost Ossetian	Ttm Tatar_Mishar	
Cvs Chuvash	Igs Ingushian	Kmk Kumyk	Pol Polish	Tts Tatar_Siberian	

Fig. S9. Geographical distribution of ancient and modern HBV of genotype B[12]. NRGB: Non-recombinant of genotype B. RGB: Recombinant of genotype B.

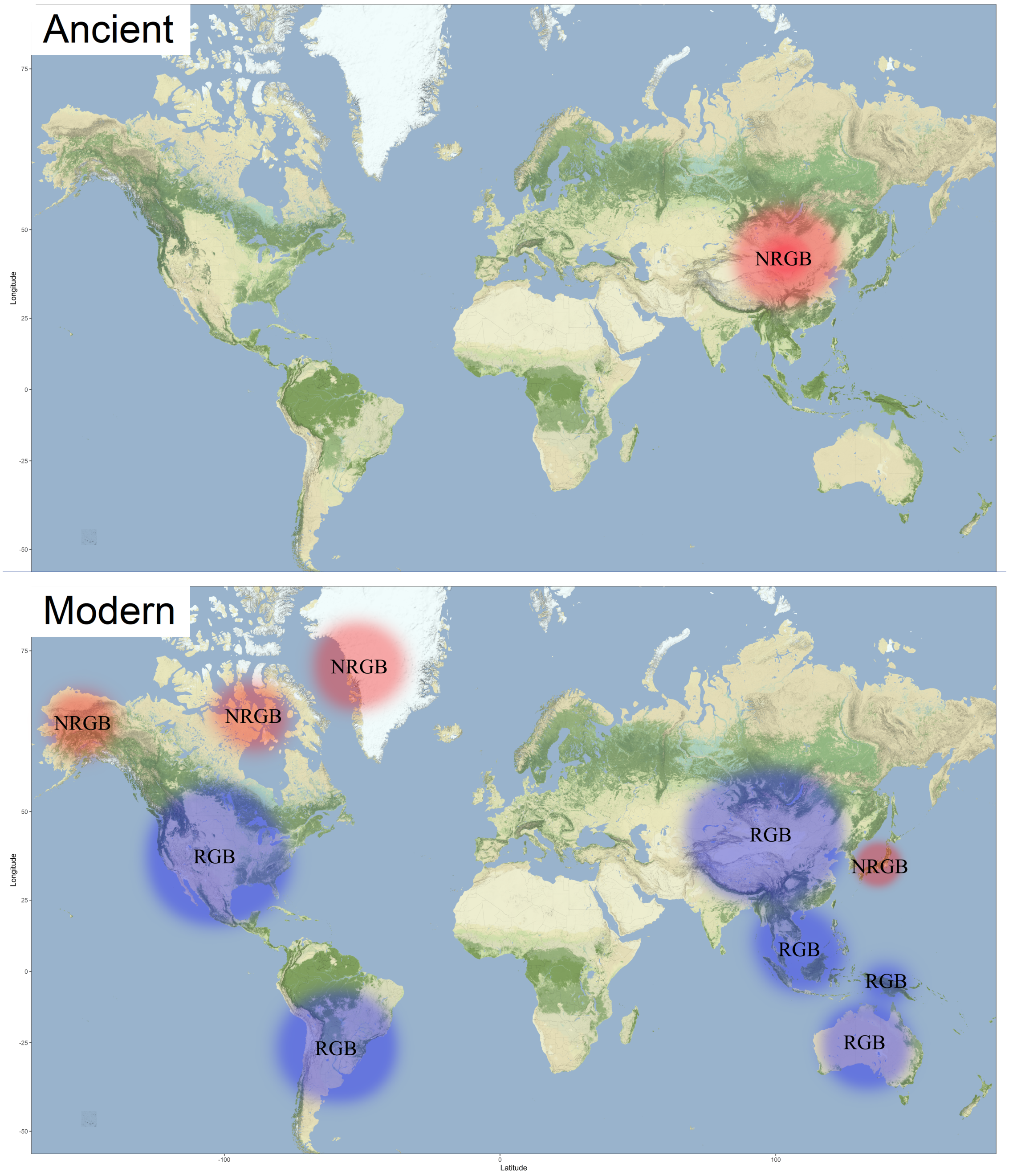

Fig. S10. Misaligned region. An additional stretch of 9 nucleotides (pos. 2990-2998) was masked due to problematic alignment as described.

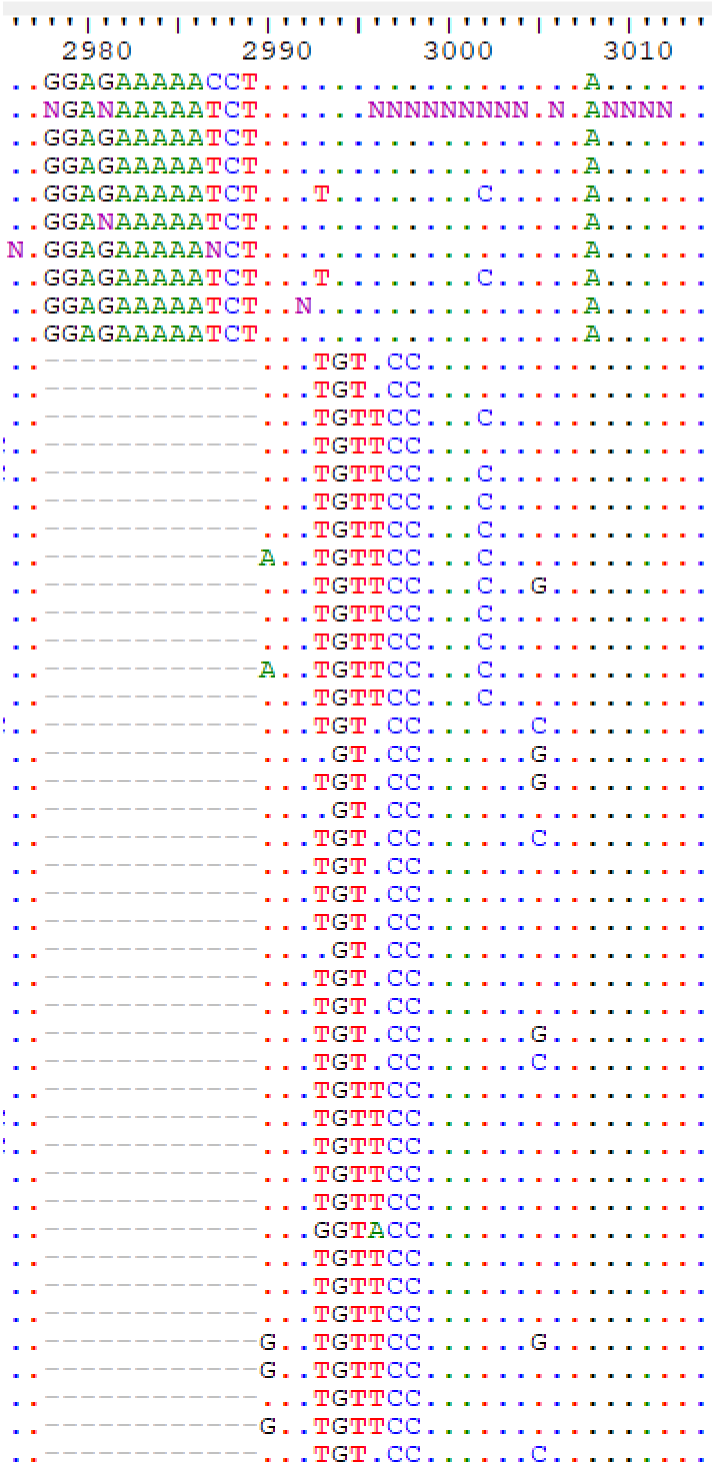

Section 4: Tables. S1 to S3

Table S1. ^{14}C dating information of HBV-positive individuals. (These ages were calibrated using OxCal v.4.4.4 (https://c14.arch.ox.ac.uk/oxcal/OxCal.html)[13] using the IntCal20 atmospheric curve[14])

Lab Code	Sample Name	^{14}C Dating (cal. a B.P.)	Calibrated Radiocarbon Age	
			1σ(68.2%)	2σ(95.4%)
ZK11594	MY12	715±20	1276AD(68.3%)1292AD	1268AD(94.2%)1300AD<br>1373AD(1.2%)1376AD
ZK11595	XN12	2060±20	98BC(32.7%)70BC<br>58BC(24.0%)39BC<br>11BC(11.5%)2AD	152BC(5.6%)130BC<br>120BC(89.8%)8AD
ZK11596	FLTM101	2155±20	344BC(25.6%)319BC<br>202BC(42.6%)164BC	350BC(33.8%)290BC<br>210BC(61.6%)102BC
ZK11597	FLTM48	2130±25	196BC(68.3%)102BC	344BC(9.0%)320BC<br>202BC(75.8%)88BC<br>80BC(10.6%)52BC
ZK11598	TJZM25_2	1765±30	244AD(14.0%)259AD<br>279AD(54.3%)335AD	230AD(95.4%)380AD
ZK11599	11KBM13	3405±30	1742BC(25.8%)1708BC<br>1700BC(24.7%)1667BC<br>1658BC(17.8%)1632BC	1868BC(3.1%)1850BC<br>1770BC(92.4%)1617BC
ZK11600	98JJLM9	4065±25	2629BC(51.4%)2568BC<br>2523BC(16.9%)2498BC	2844BC(6.5%)2812BC<br>2741BC(0.6%)2733BC<br>2674BC(65.0%)2555BC<br>2544BC(23.3%)2488BC
ZK11601	ZQM16	1580±25	435AD(27.3%)465AD<br>474AD(24.6%)502AD<br>507AD(7.4%)516AD<br>530AD(9.0%)540AD	423AD(95.4%)550AD
ZK11602	XBQM46	2885±25	1111BC(68.3%)1015BC	1196BC(3.2%)1173BC<br>1160BC(2.4%)1144BC<br>1130BC(89.2%)982BC<br>946BC(0.6%)940BC
ZK11604	95JJLM34	4130±25	2856BC(22.7%)2807BC<br>2750BC(12.9%)2723BC<br>2700BC(32.7%)2630BC	2869BC(29.0%)2801BC<br>2778BC(62.4%)2620BC<br>2606BC(4.1%)2582BC
ZK11605	95JJLM51	4130±25	2856BC(22.7%)2807BC<br>2750BC(12.9%)2723BC<br>2700BC(32.7%)2630BC	2869BC(29.0%)2801BC<br>2778BC(62.4%)2620BC<br>2606BC(4.1%)2582BC
ZK11606	XBQM125	2895±25	1118BC(58.5%)1044BC<br>1032BC(9.8%)1018BC	1201BC(11.7%)1142BC<br>1132BC(83.8%)1004BC
ZK11608	XHM18	2440±25	731BC(14.4%)699BC<br>662BC(5.9%)650BC<br>544BC(40.3%)462BC<br>438BC(7.7%)420BC	750BC(22.3%)685BC<br>667BC(9.4%)637BC<br>588BC(1.1%)578BC<br>572BC(62.6%)408BC

Table S2. Consensus sequence identity to the sequences of genotype B.

	B1_D23679.1	B2_JQ801514.1	B3_AP011085.1	B4_AB073835.1	B5_AB287314.1
96NVZIM6	0.972	0.964	0.963	0.959	**0.973**
SBSM101	**0.985**	0.972	0.954	0.963	0.959
JHM2098	0.974	0.963	0.967	0.958	**0.977**
91KLH18	0.970	0.960	0.967	0.955	**0.973**
XHM18	**0.973**	0.961	0.964	0.956	**0.973**
XBQM47	**0.982**	0.969	0.968	0.964	0.979
FLTM101	**0.987**	0.976	0.972	0.971	0.982
FLTM48	0.982	0.971	0.972	0.966	**0.983**
AT19	**0.985**	0.972	0.960	0.966	0.968
AT24	**0.975**	0.962	0.964	0.957	0.971
AT7	**0.988**	0.975	0.966	0.970	0.975
TJZM25_2	**0.986**	0.974	0.957	0.964	0.963
XN12	0.983	0.973	0.973	0.967	**0.984**
FLTM97	**0.983**	0.973	0.963	0.961	0.977
XHM12	**0.978**	0.965	0.965	0.957	0.974

Table S3. Results of phylogenetic model selection using path sampling.

tree prior	clock model	marginal likelihood estimate	logBF (in comparison to best model)
Coalescent Exponential Population	Relaxed-log	-76282.313	0
Coalescent Bayesian Skyline	Relaxed-log	-76309.7819	-27.4689139
Coalescent Constant Population	Relaxed-log	-76340.8428	-58.5297772
Birth Death Model	Relaxed-log	-76571.9171	-289.604156
Coalescent Exponential Population	Strict	-76825.9611	-543.648129
Coalescent Bayesian Skyline	Strict	-76842.0851	-559.772082
Coalescent Constant Population	Strict	-76886.8951	-604.582098
Birth Death Model	Strict	-77186.7078	-904.394778

Reference

1. McNaughton AL, Revill PA, Littlejohn M, Matthews PC, Ansari MA. Analysis of genomic-length HBV sequences to determine genotype and subgenotype reference sequences. *J Gen Virol* **101**, 271-283 (2020).

2. Zhou L, Mijiddorj E. Stories behind the fortress: Stable isotope analysis and 14C dating of soldiers' remains from the Bayanbulag site, Mongolia. *Archaeometry* **62**, 863-874 (2020).

3. Kovalev A, Erdenebaatar D, Matrenin S, Grebennikov II. The Shouxiangcheng Fortress of the Western Han Period-Excavations at Baian Bulag, Nomgon Sum, Omnogov'Aimag, Mongolia. In: *Xiongnu archaeology*) (2011).

4. Zhao YB*, et al.* Ancient DNA evidence supports the contribution of Di-Qiang people to the han Chinese gene pool. *Am J Phys Anthropol* **144**, 258-268 (2011).

5. Ning C*, et al.* Ancient genomes from northern China suggest links between subsistence changes and human migration. *Nature communications* **11**, 2700-2700 (2020).

6. Yongsheng HCGXW. Preliminary Report on the 2017 Excavation of the Qin Cemetery at Fuluta in Jungar Banner, Inner Mongolia. *Archaeology and Cultural Relics*, (2019).

7. Zhang Y*, et al.* Genetic diversity of two Neolithic populations provides evidence of farming expansions in North China. *Journal of Human Genetics* **62**, 199-204 (2017).

8. Mair VH. Epigone or Progenitor of Small River Cemetery No. 5? *Reconfiguring the silk road: New research on east-west exchange in antiquity*, 23 (2014).

9. Jónsson H, Ginolhac A, Schubert M, Johnson PLF, Orlando L. mapDamage2.0: fast approximate Bayesian estimates of ancient DNA damage parameters. *Bioinformatics* **29**, 1682-1684 (2013).

10. Stamatakis A. RAxML version 8: a tool for phylogenetic analysis and post-analysis of large phylogenies. *Bioinformatics (Oxford, England)* **30**, 1312-1313 (2014).

11. Rambaut A, Lam TT, Max Carvalho L, Pybus OG. Exploring the temporal structure of heterochronous sequences using TempEst (formerly Path-O-Gen). *Virus Evol* **2**, vew007-vew007 (2016).

12. Velkov S, Ott JJ, Protzer U, Michler T. The Global Hepatitis B Virus Genotype Distribution Approximated from Available Genotyping Data. *Genes (Basel)* **9**, (2018).

13. Bronk Ramsey C. Bayesian Analysis of Radiocarbon Dates. *Radiocarbon* **51**, 337-360 (2009).

14. Reimer PJ*, et al.* The IntCal20 Northern Hemisphere radiocarbon age calibration curve (0–55 cal kBP). *Radiocarbon* **62**, 725-757 (2020).
