# Supplementary material for: Origin and dispersal history of Hepatitis B virus in Eastern Eurasia
Source: Nat Commun. 2024 Apr 5;15:2951. doi: 10.1038/s41467-024-47358-6 (PMC10997587; doi:10.1038/s41467-024-47358-6)
Supplement: Supplementary file 3 — Description of Additional Supplementary Files [file 41467_2024_47358_MOESM3_ESM.pdf]

Description of Additional Supplementary Files

File Name: Supplementary Data 1
Description: Summary of information for the individuals from which ancient HBV genomes were recovered in this study.

File Name: Supplementary Data 2
Description: Plots used to identify mixed HBV infections in this study.

File Name: Supplementary Data 3
Description: Alignment results, including the high and low coverage sequences in this study and published sequences.

File Name: Supplementary Data 4
Description: Consensus sequence identity to the sequences of ancient sequences.

File Name: Supplementary Data 5
Description: Recombination events summary in this study

File Name: Supplementary Data 6
Description: Output file of RDP5.

File Name: Supplementary Data 7
Description: Information of published data we used.
