# Supplementary figures and images for: Origin and dispersal history of Hepatitis B virus in Eastern Eurasia
Source: Nat Commun. 2024 Apr 5;15:2951. doi: 10.1038/s41467-024-47358-6 (PMC10997587; doi:10.1038/s41467-024-47358-6)

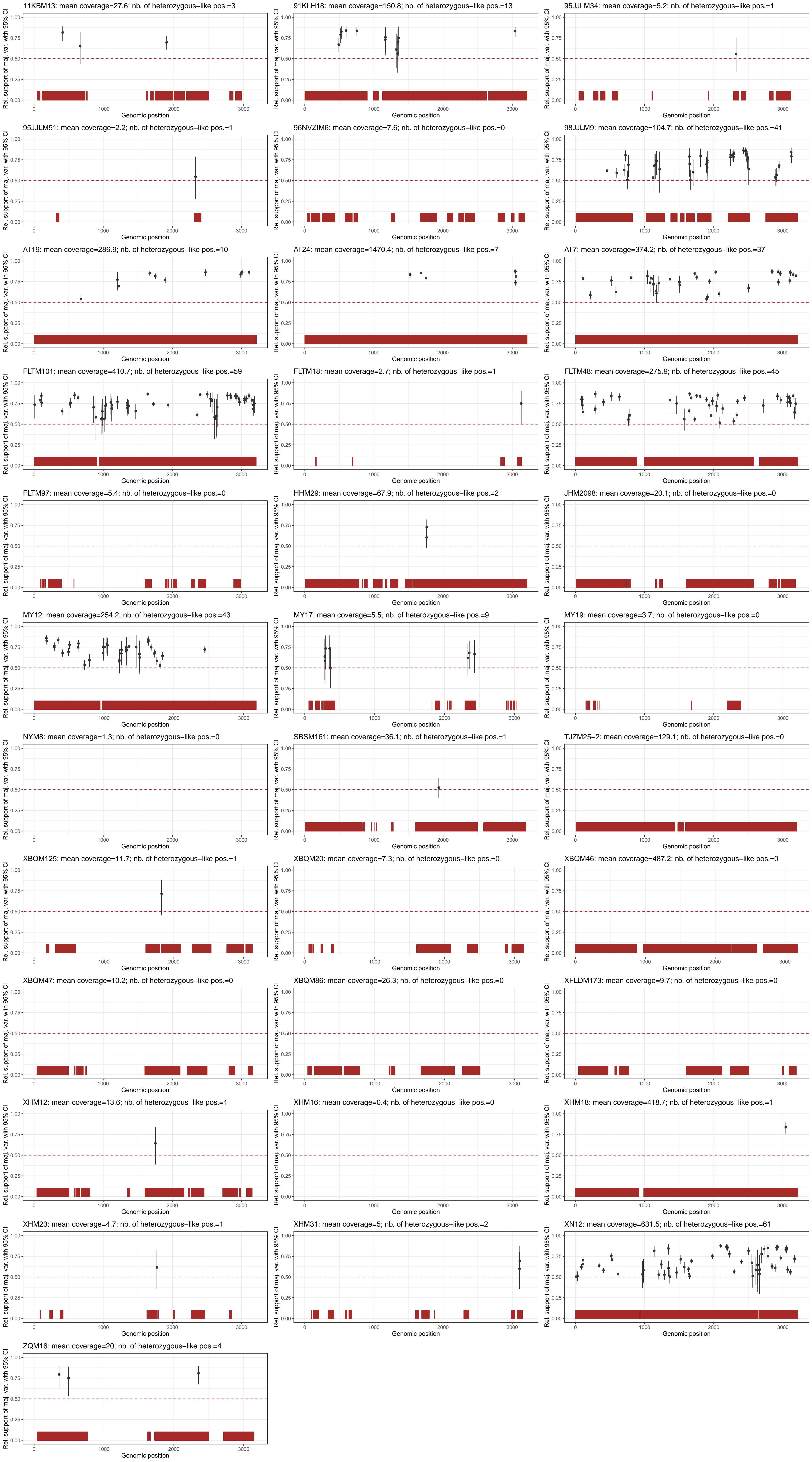

Supplement: Supplementary file 5 — Supplementary Data 2 [file 41467_2024_47358_MOESM5_ESM.pdf]
