# Supplementary material for: Origin and dispersal history of Hepatitis B virus in Eastern Eurasia
Source: Nat Commun. 2024 Apr 5;15:2951. doi: 10.1038/s41467-024-47358-6 (PMC10997587; doi:10.1038/s41467-024-47358-6)
Supplement: Supplementary file 11 — Reporting Summary [file 41467_2024_47358_MOESM11_ESM.pdf]

nature portfolio

Corresponding author(s): Yinqiu Cui

Last updated by author(s): 2024, Mar 22

Reporting Summary

Nature Portfolio wishes to improve the reproducibility of the work that we publish. This form provides structure for consistency and transparency in reporting. For further information on Nature Portfolio policies, see our Editorial Policies and the Editorial Policy Checklist.

Statistics

For all statistical analyses, confirm that the following items are present in the figure legend, table legend, main text, or Methods section.

n/a	Confirmed	
☐	☒	The exact sample size (*n*) for each experimental group/condition, given as a discrete number and unit of measurement
☒	☐	A statement on whether measurements were taken from distinct samples or whether the same sample was measured repeatedly
☒	☐	The statistical test(s) used AND whether they are one- or two-sided<br>*Only common tests should be described solely by name; describe more complex techniques in the Methods section.*
☒	☐	A description of all covariates tested
☒	☐	A description of any assumptions or corrections, such as tests of normality and adjustment for multiple comparisons
☐	☒	A full description of the statistical parameters including central tendency (e.g. means) or other basic estimates (e.g. regression coefficient) AND variation (e.g. standard deviation) or associated estimates of uncertainty (e.g. confidence intervals)
☒	☐	For null hypothesis testing, the test statistic (e.g. *F*, *t*, *r*) with confidence intervals, effect sizes, degrees of freedom and *P* value noted<br>*Give P values as exact values whenever suitable.*
☐	☒	For Bayesian analysis, information on the choice of priors and Markov chain Monte Carlo settings
☒	☐	For hierarchical and complex designs, identification of the appropriate level for tests and full reporting of outcomes
☒	☐	Estimates of effect sizes (e.g. Cohen's *d*, Pearson's *r*), indicating how they were calculated

Our web collection on statistics for biologists contains articles on many of the points above.

Software and code

Policy information about availability of computer code

Data collection	Illumina sequencing data were processed using the following programs to obtain genotype data used in the analysis: EAGER (v.1.92.37), FastQC (v.0.11.8), AdapterRemoval (v.2.1.7), bwa (v.0.7.17-r1188), samtools (v.1.3), bedtools (v.2.25.0), mapDamage (v.2.0.9-dirty), GATK (v.3.7-0-gcfedb67), TOPAS (v.1.0.1). These programs are publicly available.
Data analysis	MALT (v.0.5.3), OxCal (v.4.4), MAFFT (v.7.305b), BioEdit (v.7.2.5), RAxML (v.8.2.12), SplitsTree (v.4.19.2), TempEst (v.1.5.3), BEAST (v.2.6.6), TreeAnnotator (v.2.6.6), RDP5, SimPlot (v.3.5.1), smartpca (v.13050), PLINK (v.1.90), ADMIXTURE (v.1.3.0).

For manuscripts utilizing custom algorithms or software that are central to the research but not yet described in published literature, software must be made available to editors and reviewers. We strongly encourage code deposition in a community repository (e.g. GitHub). See the Nature Portfolio guidelines for submitting code & software for further information.

Data

Policy information about availability of data

All manuscripts must include a data availability statement. This statement should provide the following information, where applicable:

- Accession codes, unique identifiers, or web links for publicly available datasets
- A description of any restrictions on data availability
- For clinical datasets or third party data, please ensure that the statement adheres to our policy

The raw sequence data reported in this paper have been deposited in the Genome Sequence Archive in National Genomics Data Center, China National Center for

Bioinformation / Beijing Institute of Genomics, Chinese Academy of Sciences (GSA: CRA013222) that are publicly accessible at https://ngdc.cncb.ac.cn/gsa. The human reference genome used in this paper can be downloaded at ftp://ftp.1000genomes.ebi.ac.uk/vol1/ftp/technical/reference/phase2_reference_assembly_sequence/hs37d5.fa.gz. The database of ancient and present-day individuals at 1,233,013 sites and the Human Origins array with 597,573 sites can be downloaded at https://reich.hms.harvard.edu/allen-ancient-dna-resource-aadr-downloadable-genotypes-present-day-and-ancient-dna-data. The basemaps used in Fig.1, Fig.2 and Fig.S9 are in the public domain and accessible through the Natural Earth website (https://www.naturalearthdata.com/downloads/10m-raster-data/). The basemaps used in Fig. S1 is from ggplot2.

Research involving human participants, their data, or biological material

Policy information about studies with human participants or human data. See also policy information about sex, gender (identity/presentation), and sexual orientation and race, ethnicity and racism.

Reporting on sex and gender	In this study, the experimental samples utilized consist of ancient human teeth and temporal bones sourced from archaeological sites. Throughout the collection phase, every accessible sample from the sites was gathered without distinction, thus mitigating any potential sex bias.
Reporting on race, ethnicity, or other socially relevant groupings	See above
Population characteristics	See above
Recruitment	See above
Ethics oversight	See above

Note that full information on the approval of the study protocol must also be provided in the manuscript.

Field-specific reporting

Please select the one below that is the best fit for your research. If you are not sure, read the appropriate sections before making your selection.

☐ Life sciences ☐ Behavioural & social sciences ☒ Ecological, evolutionary & environmental sciences

For a reference copy of the document with all sections, see nature.com/documents/nr-reporting-summary-flat.pdf

Ecological, evolutionary & environmental sciences study design

All studies must disclose on these points even when the disclosure is negative.

Study description	In this study, we used MALT to screen 869 ancient sequence data sets to detect the presence of HBV DNA. We found 34 individuals have reads mapping to HBV. These ancient individuals from China, Mongolia and Russian Federation, ranging between 5000 to 400 BP.
Research sample	Research samples are composed of 34 ancient genomes from various archaeological sites from China, Mongolia and Russian Federation. These ancient samples were carried HBV. These 34 ancient genomes are categorized into five genotypes (A, B, C, D, WENBA).
Sampling strategy	No sample-size selection was performed prior to the study. To produce ancient genomes reported in this study, we detect the presence of HBV DNA from a wide geographic regions and time periods.
Data collection	Libraries that were prepared in Jilin were directly shotgun sequenced on an Illumina HiSeq X10 or HiSeq 4000 instrument at the Novogene company, China, in the 150-bp paired-end sequencing design. Libraries prepared at IVPP, all the samples were enriched and were sequenced on an Illumina HiSeq 4000 instrument in Beijing, China using 2x150bp chemistry. The sequencing work was completed by members of the sequencing company.
Timing and spatial scale	Laboratory works and sequencing were conducted over the period from March 2021 to September 2022. Samples were taken from various archaeological sites in China, Mongolia and Russian Federation. Detailed information of the archaeological samples studied in this manuscript is provided in Fig.1a, Supplement and Supplementary Data S1.xlsx.
Data exclusions	For phylogenetic, molecular dating and recombination analysis of the ancient HBV. We use the ancient genomes with more than 50% genome coverage to assess the phylogenetic placement of the new ancient genomes in relation to all currently known HBV diversity.
Reproducibility	Robustness of the phylogenetic inference (maximum likelihood) was tested with bootstrapping (1,000 replicates). Temporal signal for molecular dating was present in the our dataset. When repeating the construction of the ML and MCC tree, We found that only a few branches' topological structures changed, while the overall topological structure remained unchanged.
Randomization	The 869 samples screened in this study were not manually selected.
Blinding	There was no experimental treatment of samples involved in this study that requires blinding.

Did the study involve field work? ☐ Yes ☒ No

Reporting for specific materials, systems and methods

We require information from authors about some types of materials, experimental systems and methods used in many studies. Here, indicate whether each material, system or method listed is relevant to your study. If you are not sure if a list item applies to your research, read the appropriate section before selecting a response.

Materials & experimental systems

n/a	Involved in the study
☒	☐ Antibodies
☒	☐ Eukaryotic cell lines
☐	☒ Palaeontology and archaeology
☒	☐ Animals and other organisms
☒	☐ Clinical data
☒	☐ Dual use research of concern
☒	☐ Plants

Methods

n/a	Involved in the study
☒	☐ ChIP-seq
☒	☐ Flow cytometry
☒	☐ MRI-based neuroimaging

Palaeontology and Archaeology

Specimen provenance	The specimens reported in this study come from 17 archaeological sites: Tsagaan Del, XiHe, NanYang, TaoJiaZhai, QuanErGou, Derestuj, XingFuLinDai, LongTouShan, FuLuTa, JiangJiaLiang, QiLangShan, Northern, Honghe, Hengshui, Niuheliang, Tiantaijie. The archaeological context are described in the Supplementary information.<br>The ancient samples newly reported in this research were collected with the approval of the sample custodians, who are archaeologists or museums from each of the countries for which we analyzed the data. Each newly reported ancient sample in this study has received analysis permission from its custodians, who are collaborators and confirm that conducting ancient DNA analysis on these samples is appropriate.
Specimen deposition	The specimens reside under the stewardship of the archaeologists and affiliated cultural institutions from which they were sourced. They are available for re-examination upon formal request directed to the respective archaeologists.
Dating methods	Dating work was carried out in the C14 laboratory of the Center for Scientific Archaeology, Institute of Archaeology, Social Sciences of Chinese Academy. Only 14 out of 34 positive individuals have sample dates determined by C14 dating, using the same samples from which DNA was extracted. The C14 dates were calibrated using OxCal v.4.4 using the IntCal20 atmospheric curve. This is followed by the median probability calibrated age before the present (cal. a. B.P.). The dates for MY17, MY19, XHM12, XHM16, XHM23, XHM31, NYM9, AT7, AT19, AT24, XBQM20, XBQM47, XBQM86, FLTM18, FLTM97, HHM29, SBSM101, 96NVZIM6, JHM2098, and have been estimated based on the dates of other individuals from the same site. 91KLH18 has been dated before.

☒ Tick this box to confirm that the raw and calibrated dates are available in the paper or in Supplementary Information.

Ethics oversight	This study relies on archaeological remains previously excavated and incorporates neither new excavation endeavors nor research involving living human or animal subjects. Every newly reported ancient sample in this study has permission for analysis from custodians of the samples who are co-authors and who affirm that ancient DNA analysis of these samples is appropriate.

Note that full information on the approval of the study protocol must also be provided in the manuscript.
